# Structural basis for binding of human IgG1 to its high-affinity human receptor FcγRI

**DOI:** 10.1038/ncomms7866

**Published:** 2015-04-30

**Authors:** Masato Kiyoshi, Jose M.M. Caaveiro, Takeaki Kawai, Shinya Tashiro, Teruhiko Ide, Yoshiharu Asaoka, Kouta Hatayama, Kouhei Tsumoto

**Affiliations:** 1 Department of Bioengineering, Graduate School of Engineering, The University of Tokyo, 7-3-1 Hongo, Tokyo 113-8656, Japan; 2 Department of Medical Genome Sciences, Graduate School of Frontier Sciences, The University of Tokyo, Kashiwa 277-8562, Japan; 3 Tosoh Corporation, Hayakawa, Ayase 252-1123, Japan; 4 Sagami Chemical Research Institute, Hayakawa, Ayase 252-1193, Japan; 5 Laboratory of Medical Proteomics, Institute of Medical Science, The University of Tokyo, 4-6-1 Shirokanedai, Minato-ku, Tokyo 108-8639, Japan

## Abstract

Cell-surface Fcγ receptors mediate innate and adaptive immune responses. Human Fcγ receptor I (hFcγRI) binds IgGs with high affinity and is the only Fcγ receptor that can effectively capture monomeric IgGs. However, the molecular basis of hFcγRI's interaction with Fc has not been determined, limiting our understanding of this major immune receptor. Here we report the crystal structure of a complex between hFcγRI and human Fc, at 1.80 Å resolution, revealing an unique hydrophobic pocket at the surface of hFcγRI perfectly suited for residue Leu235 of Fc, which explains the high affinity of this complex. Structural, kinetic and thermodynamic data demonstrate that the binding mechanism is governed by a combination of non-covalent interactions, bridging water molecules and the dynamic features of Fc. In addition, the hinge region of hFcγRI-bound Fc adopts a straight conformation, potentially orienting the Fab moiety. These findings will stimulate the development of novel therapeutic strategies involving hFcγRI.

## Abstract (web summary)

FcγRs are cell-surface receptors for IgGs that play key roles in the humoral and cellular immune response to infection. Here, the authors present a high-resolution crystal structure of the hFcγRI-Fc complex to reveal the molecular mechanisms underlying the high specificity of this important immunological interaction.

## Graphical abstract

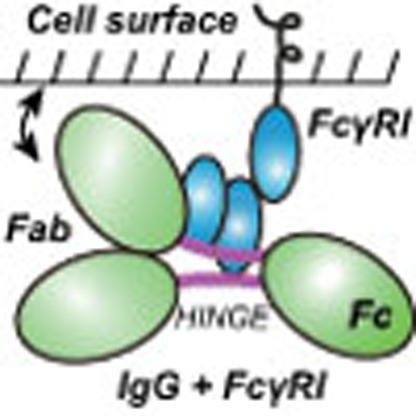

Fcγ receptors are cell-surface receptors for immunoglobulin G (IgG) that play pivotal roles in humoral and cellular protection against infection^1^,^2^. Pathogens invading the blood circulation system such as bacteria and viruses are marked for clearance by the immune system in a process known as opsonization^3^. Immune complexes are engaged by Fcγ receptors on the surface of immune cells, triggering receptor clustering and activation of multiple immune responses^4^, such as phagocytosis, antigen presentation, antibody-dependent cellular cytotoxicity, secretion of mediators and antibody production^2^,^5^,^6^.

Three different classes of human Fcγ receptors have been identified: hFcγRI (CD64), hFcγRII (types A and B, collectively known as CD32) and hFcγRIII (types A and B, collectively known as CD16). Each receptor exhibits distinctive tissue distribution, structure and binding specificity towards various IgG subclasses^7^,^8^. From a functional point of view, Fcγ receptors are divided in two classes according to their ability to activate or suppress the immune response. hFcγRI, hFcγRIIA and hFcγRIIIA are activating via the cytoplasmic immunoreceptor tyrosine-based activation motif, whereas hFcγRIIB is suppressive via signalling through the immunoreceptor tyrosine-based inhibitory motif. In addition, each Fcγ receptor exhibits distinct degrees of selectivity towards each IgG subclass (IgG1, IgG2, IgG3 and IgG4)^2^. Importantly, dysregulation of Fcγ receptors function is an important factor in several autoimmune diseases^8^,^9^,^10^,^11^,^12^, and therefore a better understanding of the molecular mechanisms involved is needed.

The hFcγRI is a major immune receptor expressed on the surface of macrophages, monocytes, neutrophils, eosinophils and dendritic cells^11^. The expression level of hFcγRI is upregulated by interferon-α and interferon-γ and by interleukin-12 (refs 13, 14, 15, 16). A large body of evidence has revealed the key roles of hFcγRI in immunity, receptor clustering, signal transduction and a connection to autoimmune diseases^13^,^17^,^18^,^19^,^20^,^21^,^22^,^23^,^24^.

Human FcγRI is a 72-kDa transmembrane glycoprotein that recruits monomeric IgG1, IgG3 and IgG4—but not IgG2—with high affinity (*K*_D_ ∼10^−9^−10^−10^ M). The specific exclusion of IgG2 is thought to be related to sequence determinants within the lower hinge of the Fc moiety (Supplementary Table 1)^25^,^26^,^27^. In contrast to receptor I, other Fcγ receptors (hFcγRIIA, hFcγRIIB, hFcγRIIIA and hFcγRIIIB) bind IgG with much lower affinity (2–3 orders of magnitude less than FcγRI) limiting their ability to capture monomeric IgG. Intriguingly, hFcγRI is the only receptor displaying a third immunoglobulin-like domain (D3) of unknown function^28^.

Although the structure of unbound hFcγRI has been recently reported^28^, no crystal structure of the relevant complex with Fc is still available, making the Fc·hFcγRI complex the last structure to be determined among the major human Fcγ cell receptors^28^,^29^,^30^,^31^,^32^,^33^. We note that the immune complex of hFcγRIIb was obtained with a version of Fc mutated at the critical hinge region, whereas the crystal structure of hFcγRIIa with Fc was determined at very low resolution 3.8 Å, thus limiting the significance of these two structures for comparative purposes. In the absence of structural information about the complex between hFcγRI and Fc, the molecular basis for their high-affinity interaction and the role of the third domain of the receptor remain unclear. In addition, atomic-level structural information is essential to a better understanding of the molecular mechanism directing immune responses, and for the development of hFcγRI-targeted therapies against autoimmune diseases^18^,^34^,^35^,^36^,^37^,^38^.

Here, we report the crystal structure of an optimized version of the ectodomain of hFcγRI in complex with human Fc at high resolution (1.80 Å). The combination of structural, kinetic and thermodynamic data reveals details of the interaction between hFcγRI and Fc with unprecedented detail. We observe unexpected differences in the conformation of the lower hinge region of Fc bound to hFcγRI with respect to that bound to other Fcγ receptors. Based on our observations, we propose that the Fab region of IgG1 may adopt a distinct orientation at the cell surface, in which domain D3 of FcγRI acts as a spacer to accommodate the Fab moieties. Overall, these results shed light on an important immunological process and should enable the development of novel therapies in the fight against autoimmune diseases in which hFcγRI is involved.

## Results

### Crystal structure of hFcγRI (CD64) bound to human Fc

**Figure 1:**
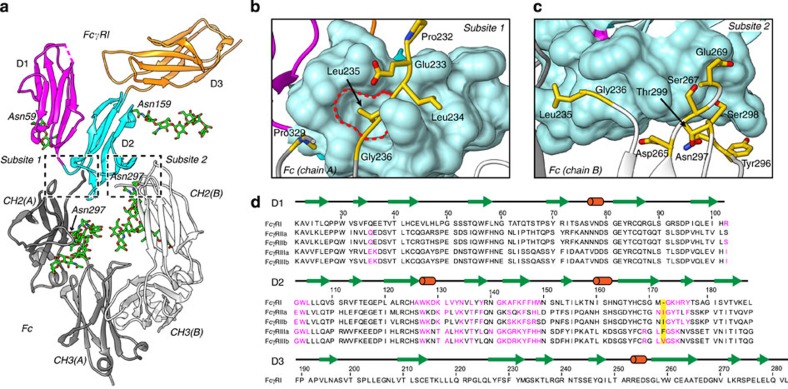
High-resolution crystal structure of the complex between hFcγRI and Fc. (**a**) Overall structure of the complex (cartoon representation). Domains D1, D2 and D3 of hFcγRI are depicted in magenta, cyan and orange, respectively. Chains A and B of Fc are shown in dark and light grey, respectively. Glycans are shown as sticks (carbon in green and oxygen in red). (**b**) Close-up view of subsite 1 (hFcγRI and chain A of Fc). The surface of D2 of hFcγRI is shown in cyan. Residues of chain A of Fc participating in the interaction with the receptor (BSA>25 Å^2^) are represented by sticks (yellow, red and blue correspond to carbon, oxygen and nitrogen, respectively). The red dashed line highlights the hydrophobic pocket where Leu235 is inserted. (**c**) Close-up view of subsite 2. Colour-code is the same as that in subsite 1. (**d**) Sequence alignment of human Fcγ receptors. The secondary structure elements cylinders (helices) and arrows (β-sheet) are indicated. Residues in magenta participate in the interaction with Fc. The yellow box indicates the position of the key residue blocking the hydrophobic pocket where residue Leu235 of Fc inserts when in complex with hFCγRI (see Fig. 4 for additional details).

We determined the crystal structure of an optimized version of hFcγRI in complex with the Fc fragment of a humanized IgG1 (rituximab) at a resolution of 1.80 Å. The data collection and refinement statistics are summarized in Table 1. The overall structure of the complex, including glycosylation sites, is shown in Fig. 1 and Supplementary Figs 1 and 2. A dimer of Fc binds asymmetrically to a molecule of hFcγRI. Residues comprising the three immunoglobulin-like domains of hFcγRI (D1, D2, D3) are clearly observed in the electron density, except for a few disordered residues at the N-terminal (16–20), the C-terminal (283–289) and a short loop within domain D1 (46–48). The refined model of Fc comprises residues 232–446 of chain A and residues 235–443 of chain B. None of the 19 mutations introduced in the sequence of hFcγRI to increase its stability and crystallizability lie within 10 Å of the Fc moiety, explaining the identical affinity of optimized and wild-type (WT) receptor for Fc (Supplementary Fig. 3).

A homodimer of Fc interacts asymmetrically with two well-defined surfaces of one molecule of hFcγRI (Fig. 1). We designated these surfaces subsite 1 (interacting with chain A of Fc) and subsite 2 (interacting with chain B of Fc; Supplementary Tables 2 and 3 and Supplementary Fig. 4). In hFcγRI, the interface in contact with Fc is composed almost exclusively of residues of domain D2. Subsite 1 consists of a rough hydrophobic surface covering 654 Å^2^ of contact interface with high shape complementarity with Fc (Sc=0.86)^39^. Subsite 1 engages Fc with numerous protein–protein as well as water-mediated H-bonds. Remarkably, two-thirds of the binding surface of subsite 1 involves interactions with residues of the lower hinge of Fc (Pro232-Ser239). Subsite 2 is flatter (Sc=0.78) and displays a smaller contact footprint with Fc (494 Å^2^). In subsite 2, the relative contribution of the lower hinge of Fc to the interaction with the receptor is modest in comparison with that in subsite 1. However, despite its smaller size, subsite 2 engages in a similar number of water-mediated and protein–protein H-bonds to that in subsite 1 (Supplementary Table 3). The glycans attached to Fc or hFcγRI make little contribution to the interaction surface as only weak van der Waals interactions are observed, specifically between GlcNAc451 of chain B of Fc and residues L136, F146 and R175 of the receptor (Supplementary Fig. 2). Although the glycans attached to Asn159 of hFcγRI do not interact with Fc within the same asymmetric unit, we note that they engage other Fc molecules through crystal lattice contacts. Overall, glycans appear to have little impact on the stabilization of the Fc·hFcγRI complex. This conclusion contrast with the very recent report of the crystal structure of hFcγRI in complex with Fc at low resolution (3.5 Å), in which it is proposed that receptor–glycan interactions are essential for achieving high affinity^40^.

### Conformational changes

**Figure 2:**
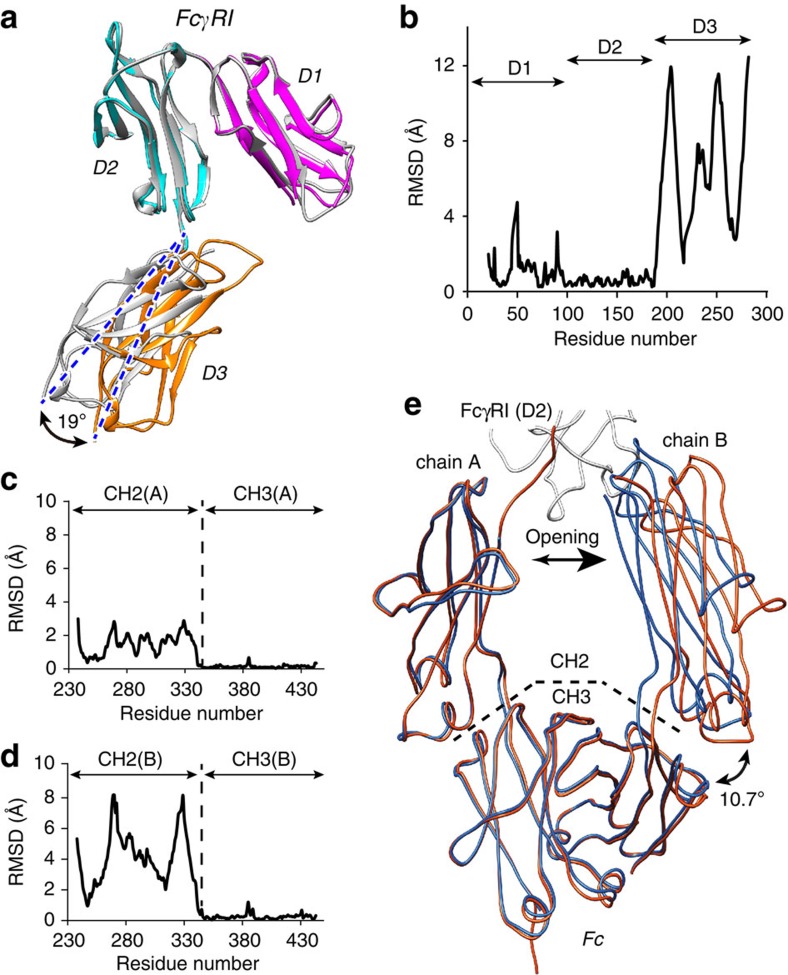
Conformational changes of hFcγRI and Fc upon binding. (**a**) Superposition of crystal structures of hFcγRI in the bound and unbound states. Domains D1, D2 and D3 of hFcγRI in the bound state are coloured in magenta, cyan and orange, respectively. Unbound hFcγRI is shown in light grey. The coordinates of unbound hFcγRI were retrieved from the PDB (entry code 3RJD). The rotation angle of domain D3 with respect to domains D1-D2 was calculated with DYNDOM^61^. (**b**) RMSD plot of bound and unbound hFcγRI. (**c**) RMSD plot of residues of chain A of Fc in the bound and unbound conformations. (**d**) RMSD plot of chain B of Fc. (**e**) Superposition of Fc in the unbound (blue ribbon) and bound (orange ribbon) conformation. Binding induces the opening of the homodimer as demonstrated by an increase of 9.1 Å in the distance between the α-carbons of Pro239 of chain A and chain B. The rotation angle between domain CH2 and domain CH3 of chain B of Fc increases by 10.7°, as calculated with DYNDOM. The dashed lines separate domain CH2 from CH3. A small portion of hFcγRI is shown at the top of the panel (light grey ribbon).

Comparison of the coordinates of hFcγRI in the bound form with the published structure of the unbound form (PDB entry code 3RJD^28^) reveals the conformational changes of the receptor upon binding of Fc. The most noticeable change occurs in the position of the uncharacterized domain D3 with respect to domains D1 and D2 (Fig. 2). Essentially, domain D3 undergoes a rigid-body displacement of up to 11 Å and a rotation of 19 ° with respect to D1 and D2 at the hinge formed by residues 185–187. Domain D3 displays the largest root-mean square deviation (RMSD) value (6.7 Å) among the three domains (calculated after superimposing domains D1 and D2, Fig. 2b). The RMSD values for D1 and D2 were 1.1 and 0.6 Å, respectively. However, pairwise comparison of each domain indicates the differences within each domain are insignificant (RMSD_D1_=0.88 Å; RMSD_D2_=0.42; RMSD_D3_=0.60 Å). Since the rigid body displacement of D3 takes place away form the binding interface, it is unclear if this movement influences the interaction with Fc or it reflects the intrinsic mobility of the receptor. We note that the different environment experienced by the receptor in different crystal lattices may influence the relative position of D3 with respect to D1–D2. No other significant change in the secondary or tertiary structure of hFcγRI is observed, demonstrating that the mutations introduced to increase its stability (ΔΔ*T*_M_=15.1°, ΔΔ*H*_DSC_=−74 kcal mol^−1^, Supplementary Fig. 3) have little impact on the local structure of the receptor.

To evaluate the conformational changes of Fc, we determined the crystal structure of the unbound protein at a resolution of 1.80 Å (Table 1). From the comparison with the bound structure, it is clear that Fc undergoes rigid-body displacement of the CH2 domains, but not the CH3 domains, upon engagement with the receptor (Fig. 2). The order of the Fc chains in the unbound and bound forms was selected in such a way that their RMSD differences were minimized when comparing the pairs chainA–chainA and chainB–chainB as described below. The most noticeable change consists in the rotation of domain CH2 of chain B of Fc with respect to the CH3 domain (10.7°), resulting in the opening of the apex of Fc near the hinge region by 9 Å (evaluated from the distance between the Cα of Pro329). This shift maximizes the molecular interactions between CH2 of chain B and subsite 2 of the receptor. In contrast, the rotation of domain CH2 of chain A is modest (amplitude was ∼2 Å calculated from the position of the Cα of Pro329).

When additional crystal structures of unbound Fc are compared with our structure, it is clear that the CH3 domains remain relatively rigid (RMSD=0.2±0.1 Å), whereas the CH2 domains are much more flexible (RMSD=2.5±1.5 Å; Supplementary Fig. 5). Importantly, the asymmetric opening of the CH2 domains of Fc upon binding to the receptor described above is far less clear when we take these additional structures into account, because the conformation of the apical region of Fc fluctuates in both chains in a similar extent. It is therefore unlikely the asymmetric mechanism of opening of chain A and B described above is of general applicability.

The analysis of the crystal structure of Fc bound to four different Fcγ receptors revealed that the fluctuations of the CH2 domain are greatly reduced when bound to receptors (RMSD_bound_=0.8±0.3 Å) with respect to the unbound state (RMSD_unbound_=2.5±1.5 Å). We note that the conformation of the CH3 domain remains constant when bound to receptors (RMSD=0.3±0.1 Å).

It has been suggested that the flexibility of the CH2 domain of Fc modulates the effector function of the immunoglobulin, highlighting the importance of these motions for the immune response^41^,^42^. The opening of Fc places each chain of Fc asymmetrically on the surface of the receptor to maximize the interaction surface and shape complementarity. In addition, several residues of the lower hinge of Fc (Pro232 to Pro238) become ordered upon binding to the receptor, suggesting this region greatly influences the recognition of the receptor by Fc (see below).

### Specific elements in the high-affinity complex hFcγRI−Fc

**Figure 3:**
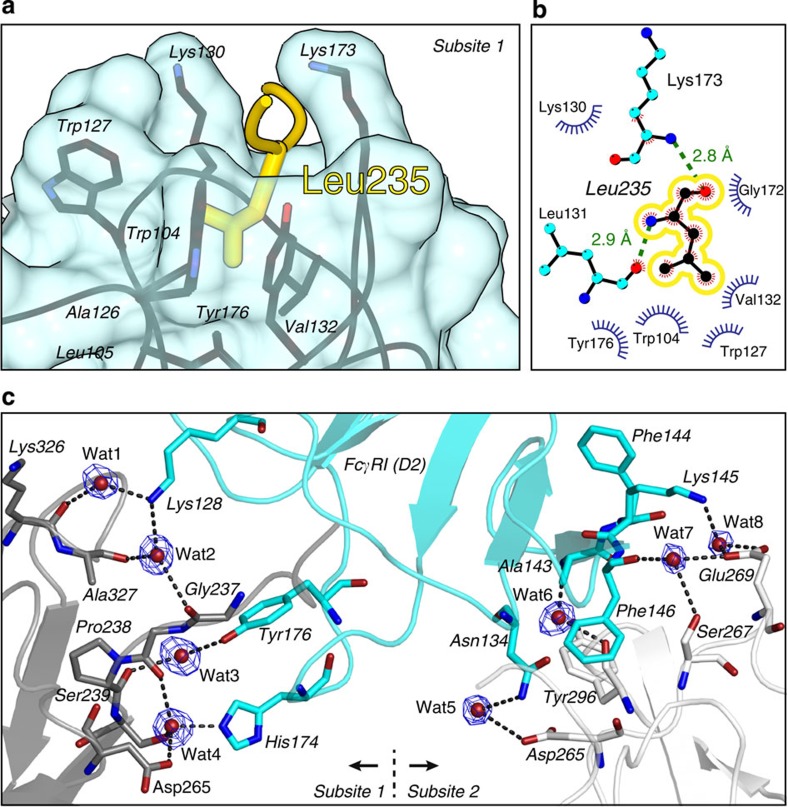
Unique recognition elements belonging to the Fc–hFcγRI complex. (**a**) Close-up view of the hydrophobic pocket of subsite 1 of hFcγRI where Leu235 of Fc inserts. Residue Leu235 is shown in yellow. The residues of hFcγRI comprising the hydrophobic pocket are depicted with black sticks. The surface of hFcγRI is shown at 70% transparency. (**b**) Schematic representation of the hydrophobic pocket. Except Leu235 of Fc, the other residues belong to domain D2 of hFcγRI. The figure was prepared with the programme LigPlot. (**c**) Water molecules bridging Fc and FcγRI. SigmaA weighted 2Fo—Fc electron density maps of the bridging water molecules (*σ*=1). The hFcγRI residues are depicted with blue sticks. Residues of chain A and chain B of Fc are depicted with dark and light grey sticks, respectively. Each water molecule engages in an average of 2.6 H-bonds with residues of the protein.

Detailed examination of the binding interface of Fc-hFcγRI reveals two distinct features not observed in the structure of Fc with other human Fcγ receptors. First, residue Leu235 of the lower hinge of Fc chain A is buried in a deep hydrophobic pocket of the receptor. This pocket is lined by residues Trp104, Lys130, Trp127, Val132, Lys173 and Tyr176 (Fig. 3 and Supplementary Fig. 6). The buried surface area (BSA) of Leu235 alone (186 Å^2^, 99% buried) accounts for 25% of the interaction surface of chain A of Fc, and suggest a critical role of this residue for binding. Previous mutational studies have suggested the importance of Leu235 for the high affinity of hFcγRI^25^,^43^,^44^. Our crystal structure clarify the molecular basis for the low affinity of hFcγRI for IgG2, as the critical residues 233–235 of IgG1 are changed in the sequence of IgG2, and therefore this region cannot interact adequately with the large hydrophobic pocket of the receptor (Supplementary Table 1)^27^. The unique characteristics of subsite 1 of hFcγRI for the high affinity of Fc could also explain the reported binding of single-chain Fc^45^.

Second, because of the high resolution and good quality of the electron density maps of our crystal structure, we identified eight well-defined water molecules bridging Fc with hFcγRI (Fig. 3 and Supplementary Fig. 4). Half of these water molecules are located at subsite 1 and half at subsite 2. These water molecules engage in an average of 2.6 H-bonds with residues of the protein. These water molecules account for far more H-bonds at the binding interface (21 hydrogen bonds) than those from direct protein–protein contacts (seven H-bonds; Supplementary Table 3), highlighting the key role of the solvent for the stability of the complex. We also note the well-conserved interaction between Pro239 of Fc and two tryptophan residues of domain D2 of hFcγRI (Trp104 and Trp127; Supplementary Fig. 4).

### Comparison with other members of the Fcγ receptor family

**Figure 4:**
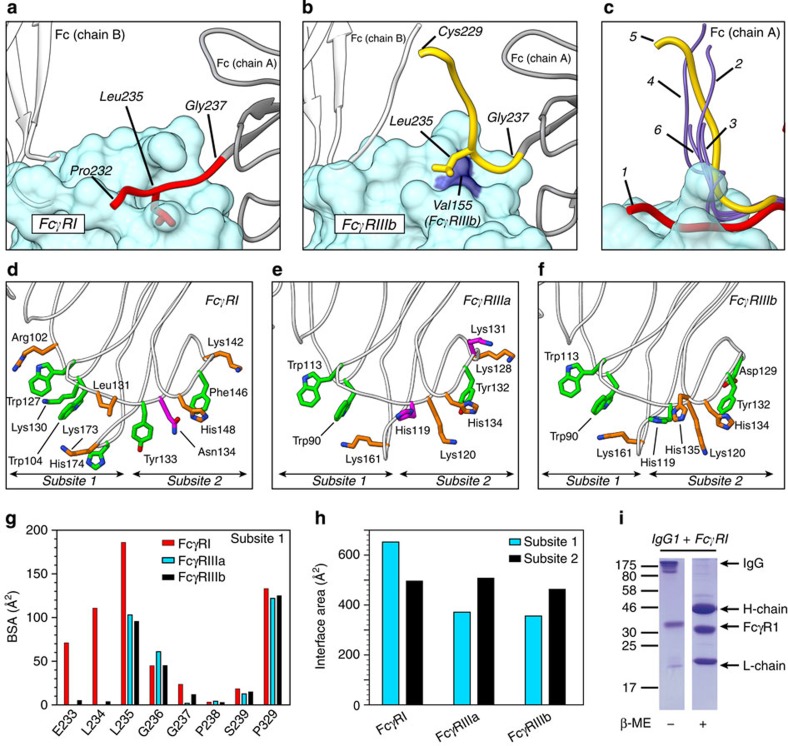
Binding of Fc to various human Fcγ receptors. (**a**) Orientation of the lower hinge of Fc (chain A, red ribbon) bound to hFcγRI (blue semi-transparent surface). Side-chain of Leu235 of Fc is shown. (**b**) Orientation of the lower hinge of Fc bound to hFcγRIIIb (PDB 1E4K). The lower hinge is depicted in yellow. The residue Val155 (dark blue surface) of hFcγRIIIb blocks the insertion of Leu235 of Fc in this receptor. (**c**) Summary of the orientations of the lower hinge of Fc in complex with Fcγ receptors retrieved from the PDB. The numbers correspond to hFcγRI (1), hFcγRIIIa (2) (PDB 3AY4), hFcγRIIIa (3) (PDB 3SGK), hFcγRIIIa (4) (PDB 3SGJ), hFcγRIIIb (5) (PDB 1E4K) and hFcγRIIIb (6) (PDB 1T83). (**d**–**f**) Key residues of Fcγ receptors present at the binding interface. Residues engaging Fc with H-bonds are depicted in orange if BSA is >40 Å^2^, and magenta if BSA <40 Å^2^. Residues displaying values of BSA >40 Å^2^, but not engaging in H-bonds with Fc, are depicted in green. Panels **d**, **e** and **f** correspond to hFcγRI, hFcγRIIIa and hFcγRIIIb, respectively. (**g**) BSA of residues of the lower hinge region of Fc in subsite 1. The plot includes Pro329 (not belonging to the hinge) for comparison purposes. (**h**) Contact interface of subsite 1 (blue) and subsite 2 (black) of various Fcγ receptors. (**i**) SDS–polyacrylamide gel electrophoresis gel of the hFcγRI–IgG1 complex under reducing and non-reducing environments. This experiment demonstrates the integrity of the disulfide bonds of the hinge region of IgG1 during our experiments.

The affinities of Fcγ receptors for the different IgG subclasses vary by nearly 1,000-fold despite their similarity at the sequence and structural levels^2^. Seeking to rationalize these differences, we compared the structure of the complex of Fc·hFcγRI with those of other Fcγ receptors deposited in the structural database. A major difference between the complex of Fc with hFcγRI and that with other human Fcγ receptors is reflected in the relative orientation of the lower hinge of Fc when bound to the receptor (Fig. 4). Whereas the unique hydrophobic pocket of hFcγRI is perfectly suited to accommodate residue Leu235 of Fc (Fig. 3), the same structural region of the other Fcγ receptors is modified by the presence of an additional hydrophobic residue in their primary sequence (yellow box in Fig. 1d). This difference is illustrated with the crystal structure of the complex of Fc with hFcγRIIIB^31^ (Fig. 4b). The additional residue Val155 of hFcγRIIIb fills up its own hydrophobic pocket, and thus blocks the putative insertion of Leu235 of Fc. As a result, the orientation of the hinge of Fc in the complex with hFcγRIIIB is different from that in the complex of Fc with hFcγRI (up and away from the receptor, forming an elbow of ∼90°). The same organization is observed in the reported structures of hFcγRIIIA^30^,^46^, and in a second structure of hFcγRIIIB^32^ (Fig. 4c). We note that in subsite 2 only few residues of the lower hinge of Fc are observed in the electron density maps. Their orientation is similar to that described in other Fcγ receptors (Supplementary Fig. 6).

Comparison of the key residues contributing to the interaction between Fc and Fcγ receptors reveals a greater number of interactions in subsite 1 of hFcγRI than in any other receptor, consistent with the important role of subsite 1 for binding (Fig. 4d–g and Supplementary Tables 2–11). Indeed, the insertion of Leu235 in the unique hydrophobic pocket of hFcγRI increases by more than threefold the collective buried surface area of the hinge residues Glu233-Leu234-Leu235 (BSA=368 Å^2^) with respect to other receptors (Fig. 4g). As a result, the ratio of the interaction surface of subsite 1 with respect to subsite 2 is 1.3 for hFcγRI, but only 0.73–0.76 for other human Fcγ receptors (Fig. 4h). Overall, the total interaction surface (subsite 1+subsite 2) in the complex of Fc with hFcγRI is the greatest among all receptors (hFcγRI=1,148 Å^2^; hFcγRIIIA=878 Å^2^; hFcγRIIIB=849 Å^2^) explaining its high affinity for IgG1.

As the disulfide bonds present in the hinge region of Fc are not observed in the crystal structures, we examined the complex between IgG1 and hFcγR1 under reducing and non-reducing conditions by SDS–polyacrylamide gel electrophoresis to verify if they remain in place during the experiments (Fig. 4i). Because IgG1 is separated in its individual chains only in the presence of the reducing agent β-mercaptoethanol, it is demonstrated that the integrity of the disulfide bond pattern remains unchanged. We also examined whether these disulfide bonds exert a significant effect on the affinity for the receptor. For that experiment we prepared a modified version of IgG1 in which the disulfide bonds of the hinge region are ablated by specific alkylation (Supplementary Fig. 7a). The binding affinity and the association/dissociation rates of Fc, IgG1 and IgG1^ALK^ to hFcγRI were evaluated by surface plasmon resonance (SPR). No significant differences were found among the three constructs assayed (Supplementary Fig. 7b–d). We conclude that the integrity of these disulfide bonds is not critical for the binding to the receptor.

### Evaluation of physicochemical parameters by SPR

**Figure 5:**
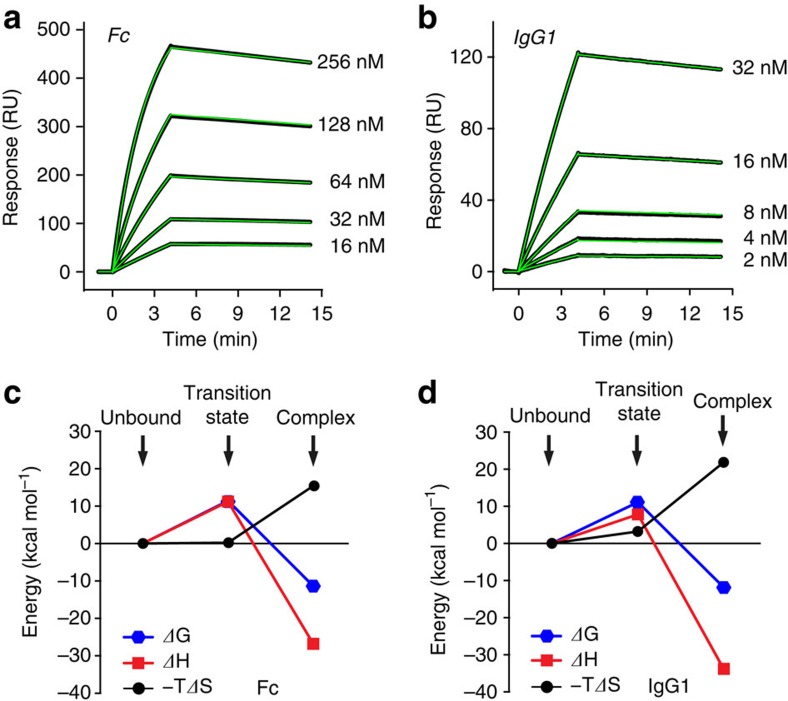
Evaluation of kinetic and thermodynamic parameters. (**a**) Sensorgrams corresponding to the binding of Fc (analyte) to a surface decorated with hFcγRI (immobilization level was ∼550 RU). The concentrations of Fc injected in each run are given. Black and green curves correspond to the experimental data and best fitting, respectively. The kinetic parameters (at 25 °C) were determined from the fitting with the software BIAevaluation (*k*_on_=2.7 × 10^4^ M^−1^ s^−1^; *k*_off_=1.1 × 10^−4^ s^−1^; *K*_D_=4.2 nM). (**b**) Sensorgrams corresponding to the binding of IgG1 to hFcγRI (immobilization level was ∼220 RU). Kinetic parameters were obtained as above (*k*_on_=4.1 × 10^4^ M^−1^ s^−1^; *k*_off_=1.2 × 10^−4^ s^−1^; *K*_D_=2.9 nM). (**c**) Evolution of the thermodynamic parameters along the reaction coordinate. Thermodynamic parameters corresponding to the transition and equilibrium states were obtained from the temperature dependence of *k*_on_ and *K*_D_ using the Eyring and van't Hoff equations, respectively. The Gibbs energy, enthalpy and entropy are shown in blue, red and black, respectively. (**d**) Thermodynamic plots for IgG1.

SPR is uniquely suited to determine a broad range of useful kinetic and thermodynamic parameters, as well as to examine the rate-limiting step (transition state) of the binding reaction. Kinetic parameters for the binding of Fc (or IgG1) to a surface decorated with hFcγRI were determined from the dose-response sensorgrams (Fig. 5). For Fc, the values of the association rate constant (*k*_on_) and the dissociation rate constant (*k*_off_) were 2.7 × 10^4^ M^−1^ s^−1^ and 1.1 × 10^−4^ s^−1^, respectively, resulting in a dissociation constant (*K*_*D*_) of 4.2 nM. These parameters are typical for high-affinity interactions. For IgG, the values of *k*_on_, *k*_off_ and *K*_*D*_ were 4.1 × 10^4^ M^−1^ s^−1^, 1.2 × 10^−4^ s^−1^ and 2.9 nM, respectively. The similarity between these values and those found for Fc demonstrates that the influence of the two Fab moieties of IgG1 is essentially negligible.

The temperature dependence of *k*_on_ and *K*_*D*_ yields useful information about the two fundamental thermodynamic states of the binding reaction coordinate: the activation (transition) state and the equilibrium state. The thermodynamic parameters of the transition state were determined from the temperature dependence of *k*_on_ (Fig. 5 and Supplementary Fig. 8)^47^. For Fc, the values of the activation free energy (*ΔG*^*‡*^=+11.4 kcal mol^−1^), activation enthalpy (*ΔH*^*‡*^=+11.2 kcal mol^−1^) and activation entropy (−*TΔS*^*‡*^=+0.2 kcal mol^−1^) demonstrate the key influence of breaking non-covalent interactions at the transition state, rather than a dynamic reorganization of solvent and/or protein. Similar observations were made when examining the binding of IgG1 (*ΔG*^*‡*^=+11.4, *ΔH*^*‡*^=+7.9 kcal mol^−1^ and *−TΔS*^*‡*^=+3.2 kcal mol^−1^).

The equilibrium parameters (van't Hoff) were obtained from the temperature dependence of *K*_*D*_ (Fig. 5 and Supplementary Fig. 8). The values of the thermodynamic parameters were consistent with the stabilization of the complex with respect to the unbound state (*ΔG*_*vH*_=−11.4 kcal mol^−1^), a process driven by the formation of numerous non-covalent interactions (*ΔH*_*vH*_=−26.8 kcal mol^−1^), and opposed by the loss of entropy (−*TΔS*_*vH*_=15.4 kcal mol^−1^). The values obtained with IgG1 led to the same conclusions, although we note that the magnitude of the entropy/enthalpy compensation was even greater for the full-length antibody (*ΔG*_*vH*_=−11.9 kcal mol^−1^, *ΔH*_*vH*_=−33.8 kcal mol^−1^ and *−TΔS*_*vH*_=22.2 kcal mol^−1^). Overall, the binding of Fc to hFcγRI displayed a greater favourable change of enthalpy than to any other receptor reported in the literature^48^.

### Interpretation of the reaction coordinate

From the examination of the thermodynamic data above, it is clear that at least two sequential processes drive the formation of the high-affinity complex between Fc and hFcγRI. First, the approach of Fc to hFcγRI involves the destruction of non-covalent forces (*ΔH*^*‡*^=+11.2 kcal mol^−1^) without a net change in the dynamics of the system (−*TΔS*^*‡*^∼0 kcal mol^−1^; Fig. 5c,d). Second, once the transition state is crossed, the complex quickly advances towards the equilibrium guided by the formation of numerous and stabilizing interactions (protein–protein and protein–solvent) but opposed by a large loss of entropy due to the ‘freezing' of side-chains and solvent molecules participating in the interaction interface.

**Figure 6:**
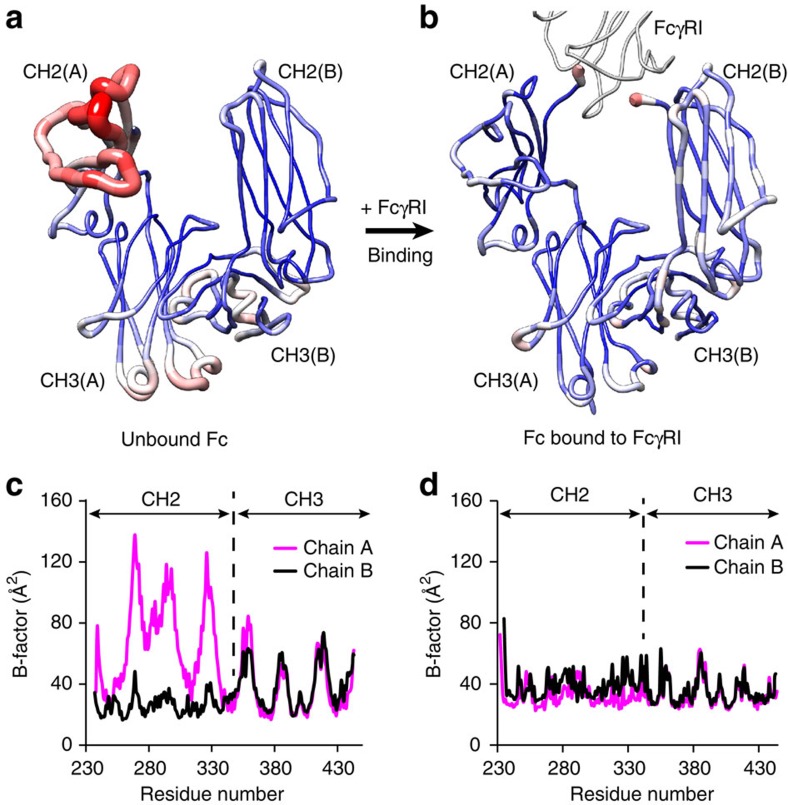
Dynamic reconfiguration of Fc upon binding to hFcγRI. (**a**) Sausage (tubular) representation of B-factors of the Fc homodimer in the unbound form. Higher B-factors are depicted by red and thick tubes. (**b**) Equivalent representation for the Fc dimer bound to the receptor. (**c**) Crystallographic B-factors of chain A (magenta) and chain B (black) of Fc in the unbound state. (**d**) Equivalent plot for the bound state. We note that the overall B-factors of each crystal structure (estimated from the Wilson plot) are very similar to each other (27.3 and 25.6 Å^2^ for the unbound and bound forms, respectively) facilitating the comparison.

We found a qualitative correlation between the dynamic changes highlighted above and the structural changes observed in Fc (Fig. 6). Crystallographic models contain dynamic information such as B-factors (temperature factors) and dynamic contact networks^49^. Thus, the comparison of crystallographic models of the bound and unbound states may reveal regions of altered dynamics in the proteins. In the case of Fc, the comparison is straightforward, as the resolution of the crystal structures in the bound and unbound form is identical (1.80 Å), and the values of the overall Wilson B-factor are very similar to each other (27.3 and 25.6 Å^2^ for unbound and bound state, respectively; Fig. 6). B-factor and tubular (sausage) representations identify the apical region of chain A of Fc (residues 265−273, 291−299 and 323−329) being the most disordered in the unbound form (B-factors>100 Å^2^). Moreover, the electron density of the lower hinge (residues before Pro238) cannot be modelled, indicating it is disordered in the crystal structure and probably in solution. On the contrary, the B-factors of Fc in the complex with hFcγRI are relatively uniform throughout the entire sequence. These two observations are consistent with the dynamic model inferred from the thermodynamic data. We note that no significant differences were found between the B-factor profiles of bound/unbound hFcγRI (Supplementary Fig. 9).

Interestingly, a similar pattern of flexible and asymmetric ensembles is observed in other crystal structures of Fc in the unbound form, except that of entry code 1LX6 (in that structure Fc is bound to a fragment of protein A; Supplementary Fig. 10). These examples include not only four additional crystal structures of Fc at high resolution in the same space group as the one we reported herein (P2_1_2_1_2_1_), but also crystal structures determined in two other different space groups (P6_1_22 and C2). Although crystal-packing forces are likely to play a role in the conformation adopted by the Fc chains, these observations suggest that the Fc portion of IgG may exist as a dynamic (flexible) and asymmetric ensemble in solution, as recently reported by other groups^42^. Given that the binding to receptors involves an asymmetric surface, the propensity of Fc to adopt flexible (asymmetric) conformations may have an impact in the binding mechanism to hFcγR1 and other Fcγ receptors.

## Discussion

We report the high-resolution crystal structure of the complex between hFcγRI and human Fc. Structural, kinetic and thermodynamic data reveal unprecedented details of the binding mechanism, and a high degree of complexity not expected from previous studies with homologous receptors. Protein–protein interactions, interfacial waters and dynamic as well as asymmetric features explain the high affinity and specificity of the Fc homodimer for hFcγRI. These are timely observations, as it has been proposed that the structural heterogeneity of Fc (IgG subtype, conformational ensemble of the apical region^42^ and variability of the composition of the glycan attached to Asn297 (ref. 41)) regulates its effector function in innate and adaptive immunity^50^.

The first type of evidence suggesting that asymmetric features of Fc may influence its binding to hFcγRI is related to the distinct organization and dynamics of each of the chains of Fc in the unbound state (Fig. 6 and Supplementary Fig. 10). Numerous crystal structures have shown that the two chains of Fc do not display symmetrical features in the crystal form (a comprehensive list can be found elsewhere^41^). This hypothesis is further supported by evidence from molecular dynamic simulations^42^. These observations collectively suggest that different conformational states could be involved during recognition of the repertoire of Fcγ receptors under various cellular environments and cell-types.

Second, binding of Fc to the surface of the receptor involves an asymmetric surface, a property that is common to all Fcγ receptors. In other words, Fc must use a different set of residues from each chain of the homodimer to engage the receptor. In subsite 1, the apical region of Fc resembles a lock (hook) governed by the insertion of Leu235 in a unique hydrophobic pocket of hFcγR1. In subsite 2, however, the interaction surface is relatively flat and does not involve local conformational changes. Given the similarities between the contact interface of Fc engaging subsite 2 of hFcγRI to that engaging subsite 2 in other Fcγ receptors, we propose this region is the common recognition platform for all receptors. In contrast, the residue Leu235 of Fc that interacts with subsite 1 represents the high-affinity element specific for hFcγRI.

**Figure 7:**
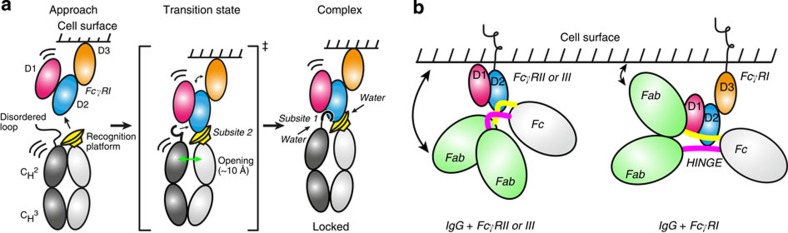
Binding model. (**a**) Stepwise mechanism of binding of Fc (IgG1) to its high-affinity cell receptor hFcγRI. The CH2 domain of one chain displays a recognition platform for its approach to subsite 2 of hFcγRI. The hinge region of the second chain of Fc still remains in a highly dynamic conformation. In the transition state the recognition platform is approaching the receptor at subsite 2, but the complex remains in a highly dynamic state. The high-affinity complex is locked when the chains of Fc open to adjust their position to the asymmetric surface of the receptor, and Leu235 of Fc inserts in the unique pocket of subsite 1, contributing to the characteristic slow dissociation rate of this complex. Multiple bridging water molecules stabilize the complex. (**b**) Model of IgG1 bound to the high-affinity receptor on the cell surface. We propose the straight conformation of the hinge region keeps the Fab moiety in a unique conformation, perhaps towards the cell surface. This conformation is enabled by domain D3, which increases the space available between the binding interface of hFcγRI and the plasma membrane.

These ideas are represented schematically in Fig. 7a. Fc exists as an ensemble in equilibrium with other symmetric and asymmetric dimers that interact with hFcγRI in a stepwise manner. The initial approach of Fc to the cell surface receptor could be guided by the invariant ‘recognition platform' common for all Fcγ receptors engaging subsite 2 of hFcγRI. Docking of this recognition platform fulfils the dynamic conditions imposed by the energetic profile (Fig. 5). The complex is locked into a stable conformation (high-affinity state) after the insertion of Leu235 of Fc in the hydrophobic pocket of subsite 1 of hFcγRI.

Importantly, the crystal structure of the immune complex with hFcγRI reveals a unique orientation of the lower-hinge residues of Fc compared with that in other Fcγ receptors (Fig. 7b). In contrast to the elbow-like conformation of the lower-hinge observed when Fc binds to other Fcγ receptors, Fc adopts a linear conformation when bound to hFcγRI. We consider this observation a significant difference that may impact the orientation of the Fab moiety of IgG1 when bound to the receptor on the cell surface. As domain D3 of the receptor does not play a direct role on the interaction with the immunoglobulin, we suggest that D3 acts as a spacer enabling the approach of the Fab moiety towards the plasma membrane. Although this hypothesis remains untested, we propose that this conformation may affect the ability of the Fab paratopes to engage with their corresponding antigens and/or facilitate the clustering of receptors on the membrane surface, perhaps modulating the effector responses mediated by hFcγRI^17^.

In summary, we determined the recognition mechanism between human IgG1 and the major cell-surface receptor hFcγRI from structural, kinetic and thermodynamic standpoints. This is an important step for the understanding of a key effector of the immune system that may guide therapeutic efforts to combat autoimmune diseases.

## Methods

### Protein cloning, expression and purification of hFcγRI

The protein sequence of WT and optimized receptor is shown in Supplementary Note 1 (ref. 51). The list of mutations is given in Supplementary Fig. 3. The construct of hFcγRI used for crystallization comprises residues Gln16 to Val289 of the optimized receptor and a hexa-histidine tag at the C-terminus. This construct was transformed in competent DH10 Bac *Escherichia coli* cells to generate a baculovirus shuttle vector (bacmid), which was subsequently transfected into Sf9 insect cells (1.0 × 10^6^ cells per ml, 2 ml) using Lipofectamine 2000 reagent (Invitrogen). After 4 days, the primary baculovirus (P1) was used to infect fresh Sf9 insect cells (1.0 × 10^6^ cells per ml, 50 ml). Two days later, the amplified (high-titre) baculovirus (P2) was collected from the infected Sf9 cells and used for protein expression.

Sf9 mimic cells incubated in Sf-900 III SF medium (Gibco) supplemented with 10% (v/v) fetal bovine serum at a density of 1.8 × 10^6^ cells per ml at 27 °C were infected by recombinant viruses (P2) at a concentration of 4% (v/v). After 4 days incubation, the cells were harvested, centrifuged at 5,800*g* and the supernatant applied onto a complete His-Tag purification resin (Roche) equilibrated with buffer A (20 mM Tris-HCl, 0.5 M NaCl, at pH 8.0). Protein was eluted by increasing of concentration of imidazole stepwise (5, 10, 20, 30, 50, 100, 200 and 300 mM) in buffer A. The fractions containing hFcγRI were pooled and dialysed against buffer B (50 mM MES, 0.1 M NaCl, pH 5.6). The dialysed sample was subjected to cation exchange chromatography in an AKTA system (GE Healthcare). The protein was eluted in a gradient of NaCl between 0.1 and 1.5 M.

### Preparation of Fc

The humanized IgG1 monoclonal antibody Rituximab was purchased from Chugai Pharma. The Fc fragment of the IgG1 monoclonal antibody was obtained by papain digestion (Pierce). Digested Fc was purified with a Protein A kit (Pierce) following the instructions of the manufacturer. The fractions containing Fc were pooled, and subjected to size exclusion chromatography using a HiLoad 26/60 Superdex 200-pg column (GE Healthcare) equilibrated with 40 mM Tris-HCl (pH 7.4) and 300 mM NaCl.

### Crystallization of Fc–FcγRI complex

Purified Fc was mixed with hFcγRI (molar ratio was 1:1), followed by dialysis overnight against 0.1 M NaCl, 20 mM Tris-HCl (pH 7.4). The Fc–hFcγRI complex was subjected to size exclusion chromatography using a HiLoad 26/60 Superdex 200-pg column (GE Healthcare) equilibrated with 20 mM Tris-HCl, 100 mM NaCl (pH 7.4) to separate the unbound protein. The fractions containing the complex were pooled and concentrated to 5.0 mg ml^−1^. The initial crystallization screening was carried out using an Oryx8 protein crystallization robot (Douglas Instruments). Single crystals were obtained in a solution of 0.1 M sodium acetate, 0.1 M zinc acetate, 4% (v:v) 1,4-butanediol and 12% PEG 4,000 (pH 4.6).

Suitable crystallization conditions for Fc at 2.5 mg ml^−1^ in 10 mM Tris-HCl (pH 7.4) were screened using commercial kits (Hampton Research). After a brief optimization, the best single crystals were prepared in a solution composed of 0.2 M calcium acetate, 0.1 M sodium cacodylate (pH 6.1) and 19% (w:v) PEG 8,000 at 20 °C.

### X-ray data collection and structure determination

Suitable crystals of the Fc–FcγRI complex or Fc were harvested, immersed in crystallization solution supplemented with 20% glycerol and frozen by submersion in liquid nitrogen. Data collection was carried out at beamline BL5A at the Photon Factory (Tsukuba, Japan) at a wavelength of 1.000 Å and a temperature of 100 K. Diffraction images were processed with the programme MOSFLM^52^. Data were merged and scaled with the programme SCALA^53^. The structure of the complex between Fc and hFcγRI was determined by the method of molecular replacement with the programme PHASER^54^ using the coordinates of Fc (PDB entry code 1L6X)^55^ and those of unbound hFcγRI (PDB entry code 3RJD)^28^. The crystallographic models were refined with the programmes REFMAC^56^ of the CCP4 suite, and manually improved in COOT^57^. The quality of the refined structure was assessed with the programmes COOT and PROCHECK^58^. No outliers in the Ramachandran plot were observed. The structure of Fc was also determined by the method of molecular replacement using the coordinates of Fc (PDB entry code 1L6X)^55^. Refinement and validation were performed as above. No outliers in the Ramachandran plot were observed.

### Preparation of alkylated IgG1

To prepare alkylated IgG1^ALK^, antibody at 3.5 μM was first incubated with 10 mM dithiothreitol at 37 °C for 1 h, followed by treatment with 50 mM iodoacetamide at 37 °C for 30 min. The reagents were eliminated by dialysis with PBS supplemented with 0.005% Tween-20. Alkylation was corroborated by non-reducing SDS–polyacrylamide gel electrophoresis.

### SPR (method #1)

The interaction between hFcγRI and Fc or IgG was analysed by SPR in a Biacore T200 instrument (GE Healthcare). Two methods were used. In the first one, proteins were dialysed against running buffer (PBS supplemented with 0.005% Tween-20). A NTA sensor chip (GE Healthcare) was used for immobilization of hFcγRI. Before each run, the NTA sensor chip was treated with 500 μM nickel chloride dissolved in running buffer at 10 μl min^−1^ for 1 min to load the sensor chip with Ni^2+^ ions. Subsequently, hFcγRI displaying a hexa-histidine at the C-terminus was immobilized on the surface chip at a density of ∼550 RU (when examined with Fc) or 220 RU (when examined with IgG). Sensorgrams corresponding to the binding of Fc or IgG1 to hFcγRI were obtained by injecting increasing concentrations of analytes at a flow rate of 30 μl min^−1^. Contact and dissociation time were 250 and 600 s, respectively. Regeneration was carried after completion of each sensorgram by injecting a solution of 0.35 M EDTA.

Data analysis was performed with the BIAevaluation software (GE Healthcare). Association (*k*_on_) and dissociation (*k*_off_) rate constants were calculated by a global fitting analysis assuming a Langmuir binding model and a stoichiometry of (1:1). The dissociation constant (*K*_D_) was determined from the ratio of the rate constants.


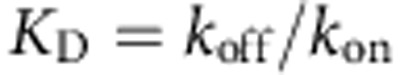


### SPR (method #2)

Kinetic parameters of the binding between hFcγRI and Fc, IgG or IgG^ALK^ were determined by SPR in a Biacore T200 instrument (GE Healthcare), using a second procedure. In this case, research grade CM5 sensor chip (GE Healthcare) was activated by a short treatment with *N*-hydroxysuccinimide and *N*-ethyl-*N′*-(3-dimethylaminopropyl) carbodiimide hydrochloride as described elsewhere^59^. The hFcγRI was immobilized at a density of 1,000 RU. Activated groups on the surface of the sensor were subsequently blocked by injecting 100 μl of a solution of 1 M ethanolamine. The kinetic data of the binding of analyte (Fc, IgG, IgG^ALK^) to FcγRI were obtained by injecting increasing concentrations into the sensor chip at a flow rate of 30 μl min^−1^. The measurements were carried out in the same running buffer. Contact time and dissociation time were 200 and 600 s, respectively. Data analysis was performed as described in method 1.

### Calculation of thermodynamic parameters using SPR

Changes in enthalpy (*ΔH*_vH_) and entropy (*ΔS*_vH_) were calculated from the slope and intercept, respectively, of the temperature dependence of the dissociation constant using the van't Hoff approximation^59^:


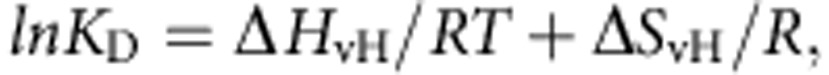


where *R* is the gas constant and *T* is the absolute temperature.

The activation energy parameters were obtained from the temperature dependence of each kinetic rate constants according to Eyring equation^60^:


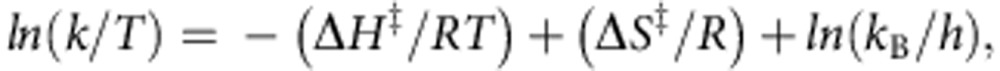


where *k* is the kinetic rate constant, *ΔH*^*‡*^ is the activation enthalpy, *R* is the gas constant, *T* is the absolute temperature, *ΔS*^*‡*^ is the activation entropy, *k*_B_ is the Boltzmann's constant and *h* is the Plank's constant. The kinetic rate constants *k*_on_ and *k*_off_ were used for the estimation of the association and dissociation activation parameters during the association and dissociation phases, respectively.

### Calorimetry

The thermal stability of hFcγRI WT and mutein was determined by differential scanning calorimetry (DSC). Each protein was concentrated to 10 μM in PBS buffer. Data collection was performed in a VP-DSC instrument (Microcal) at a scanning rate of 1.0 °C min^−1^ between 20 and 110 °C (Supplementary Fig. 3).

The affinity of IgG1 for WT and optimized hFcγRI was examined by isothermal titration calorimetry (ITC) in an VP-ITC instrument (Microcal; Supplementary Fig. 3). For this experiment, WT hFcγRI was expressed from Chinese hamster ovary cells. The protein was purified using immobilized metal affinity chromatography as described above with insect cells. The optimized hFcγRI was expressed in *E. coli* BL21 (DE3). Protein expression was induced by addition of 0.5 mM isopropyl β-D-1-thiogalactopyranoside. The cells were harvested and lysed by the sonication method. The supernatant was collected and purified using immobilized metal affinity chromatography.

Receptor and IgG1 were dialysed in PBS. The solution containing IgG1 was placed in the injection syringe at 5–8 μM. The cell contained hFcγRI (WT or mutein) at 50 μM. The volume of each injection was 10 μl except the first injection was set at 3 μl. The binding enthalpy (Δ*H°*), equilibrium-binding constant (*K*_A_) and binding stoichiometry (*n*) were determined with ORIGIN7.

## Author contributions

M.K. designed the experiments, conducted the research, analysed the data and wrote the paper. J.M.M.C. designed the overall study, designed the experiments, conducted the research, analysed the data and wrote the paper. T.K., S.T., T.I., Y.A. and K.H. designed the experiments and conducted the research. K.T. designed the overall study, designed the experiments and analysed the data.

## Additional information

**Accession codes:** The coordinates and structure factors for the complex of Fc–FcγRI and for Fc have been deposited in the Protein Data Bank under accession codes 4W4O and 4W4N, respectively.

**How to cite this article:** Kiyoshi, M. *et al.* Structural basis for binding of human IgG1 to its high-affinity human receptor FcγRI. *Nat. Commun.* 6:6866 doi: 10.1038/ncomms7866 (2015).

## Supplementary Material

Supplementary Information Supplementary Figures 1-10, Supplementary Tables 1-11, Supplementary Note 1 and Supplementary References

## Figures and Tables

**Table 1:** Data collection and refinement statistics*.

	**Fc+hFcγRI**†	**Fc**†
*Data collection*		
Space group	C2	P 2_1_ 2_1_ 2_1_
*Cell dimensions*		
*a*, *b*, *c* (Å)	134.98, 126.50, 71.61	49.71, 79.29, 138.67
α, β, γ (°)	90.0, 118.9, 90.0	90.0, 90.0, 90.0
Resolution (Å)	39.7–1.80 (1.90–1.80)	22.2–1.80 (1.90–1.80)
*R*_merge_ (%)	7.4 (87.2)	6.2 (78.7)
*I*/σ*I*	14.9 (2.3)	18.4 (2.4)
Completeness (%)	98.6 (97.4)	99.1 (97.3)
Redundancy	7.3 (7.3)	6.6 (6.4)
		
*Refinement*		
Resolution (Å)	39.7–1.80	22.2–1.80
No. reflections	95,655	51,117
*R*_work_/*R*_free_	17.3/21.5	20.0/24.4
No. of atoms		
Protein	5,475	3,354
Glycans/others‡	374	200
Water	572	377
*B*-factors		
Protein	41.5	43.6
Glycans/others‡	55.9	46.7
Water	46.0	38.1
*RMSD*		
Bond lengths (Å)	0.021	0.020
Bond angles (°)	2.1	2.2

RMSD, root-mean square deviation.

^*^Values in parentheses are for highest-resolution shell.

^†^A single crystal was employed to determine each structure.

^‡^Includes glycans covalently bound to the proteins, small ions and glycerol molecules.
