# Supplementary material for: Structural basis for binding of human IgG1 to its high-affinity human receptor FcγRI
Source: Nat Commun. 2015 Apr 30;6:6866. doi: 10.1038/ncomms7866 (PMC4423232; doi:10.1038/ncomms7866)
Supplement: Supplementary Information — Supplementary Figures 1-10, Supplementary Tables 1-11, Supplementary Note 1 and Supplementary References [file ncomms7866-s1.pdf]

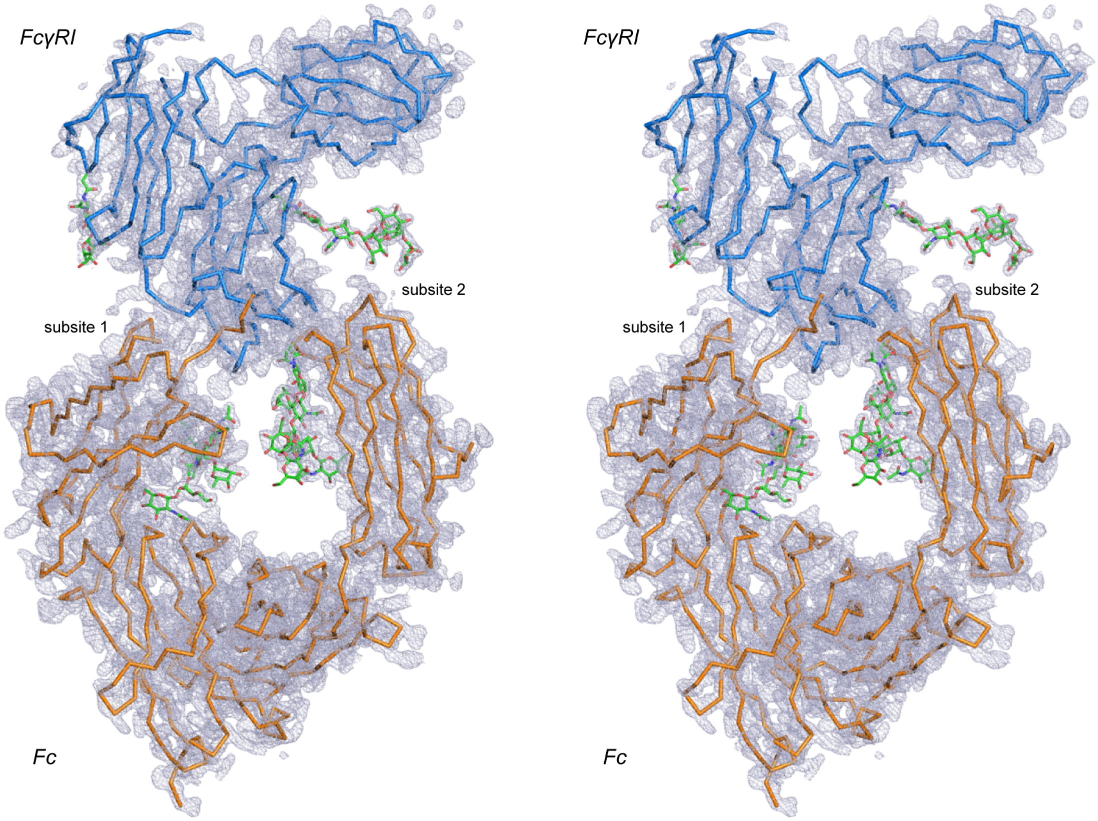

Supplementary Figure 1. Structure of human Fc bound to its high-affinity receptor hFcγRI. Stereoview of the sigmaA weighted (2Fo − Fc) electron density map of Fc bound to hFcγRI (contouring level was $\sigma = 1$). The α-carbon traces of hFcγRI and Fc are depicted in blue and orange ribbons, respectively. Subsite 1 and subsite 2 are indicated. The oligosaccharide moieties are depicted with green C-C bonds.

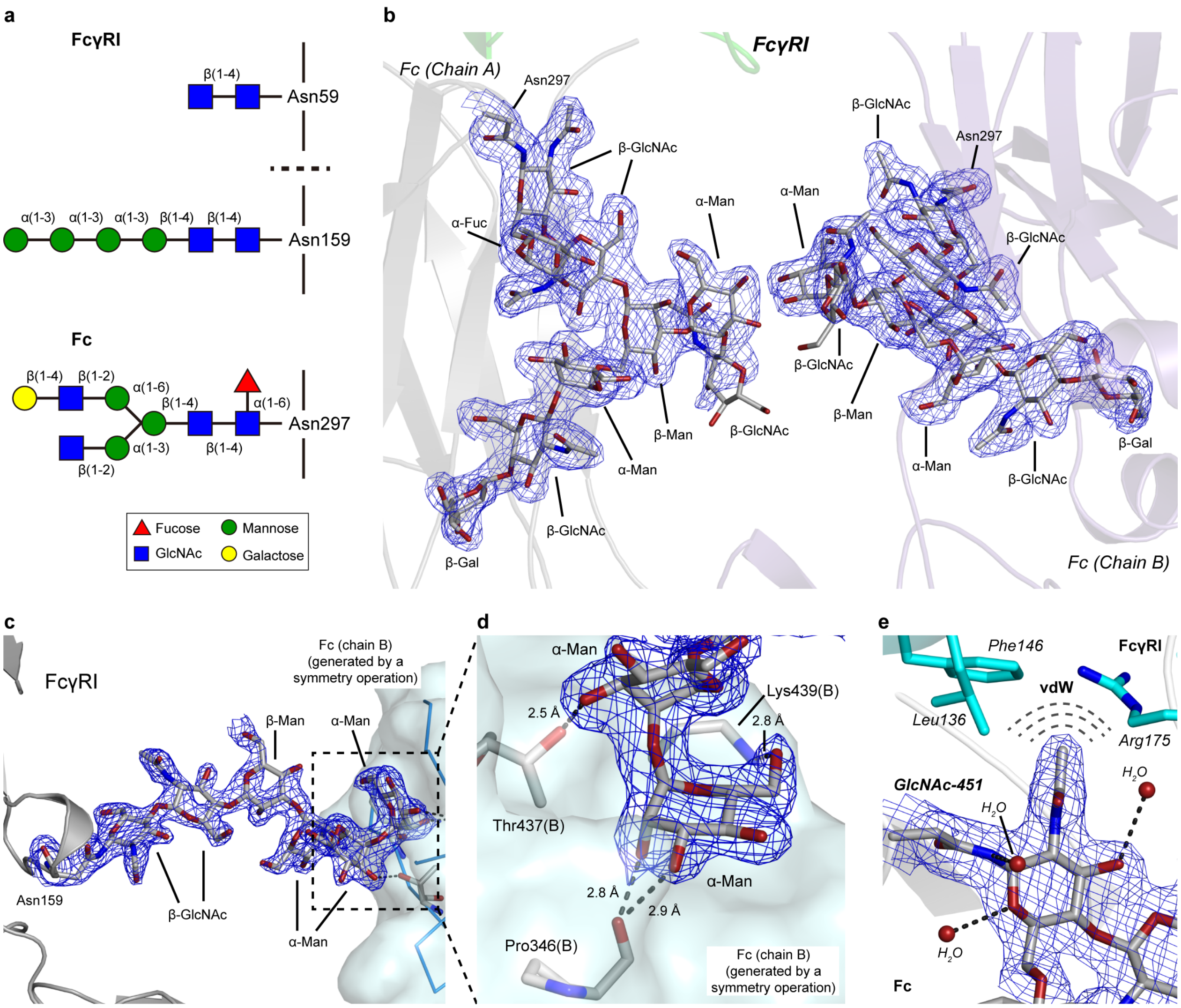

Supplementary Figure 2. Glycosylation sites.

(a) Scheme showing the oligosaccharides in hFcγRI and in Fc. All sugars are found in the stable chair conformation, except the second GlcNAc of the glycan bound to Asn59 of hFcγRI, which displays a half boat/disordered conformation. **(b)** SigmaA weighted (2Fo − Fc) electron density map of the biantennary glycans of Fc (σ = 1). **(c)** SigmaA weighted (2Fo − Fc) electron density map of the oligosaccharide bound to Asn159 of hFcγRI. The glycan is stabilized by crystal contacts with chain B of a symmetry-generated Fc molecule (light-blue surface; σ = 1). **(d)** Close-up view of the crystal contacts in (c). The orientation of the panel is rotated with respect to panel (c) to observe the H-bond interactions with more clarity. **(e)** SigmaA weighted (2Fo − Fc) electron density map of GlcNAc451 of Fc (white sticks) weakly interacting with residues of hFcγRI (blue sticks; σ = 1).

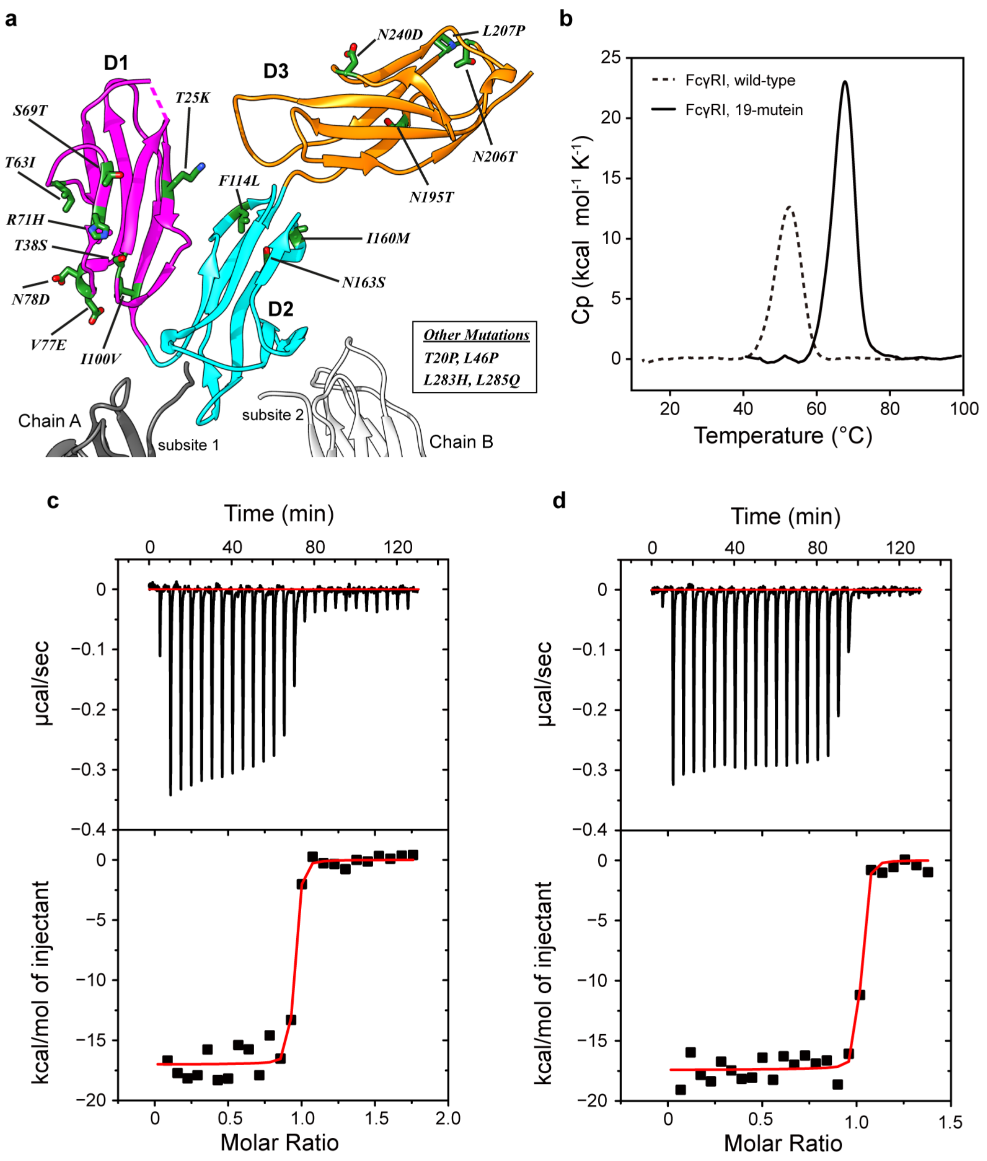

Supplementary Figure 3. Comparison between WT hFcγRI and optimized hFcγRI.

(a) Mutated residues in the optimized receptor hFcγRI (depicted in green). Mutations not shown (T20P, L46P, L283L, and L285Q) are located in disordered loops distant from the binding interface. **(b)** Thermal stability was examined by DSC (WT; T_M = 52.3 ± 0.02 °C, and ΔH_{DSC} = 105.4 ± 0.3 kcal mol^{-1}; optimized hFcγRI, T_M = 67.4 ± 0.1 °C, and ΔH_{DSC} = 180 ± 2 kcal mol^{-1}). **(c)** Titration and binding isotherm of WT receptor with IgG1. The thermodynamic parameters indicate high affinity (K_D = 1.2 nM; $\Delta H°$ = −17.0 ± 0.3 kcal mol^{-1}; $-T\Delta S°$ = 4.8 kcal mol^{-1}; $\Delta G°$ = −12.2 kcal mol^{-1}, n = 0.92 ± 0.1). **(d)** Data for the optimized hFcγRI with IgG1. The values obtained with the optimized receptor were essentially identical to those with WT receptor (K_D = 0.90 nM; $\Delta H°$ = −17.4 ± 0.3 kcal mol^{-1}; $-T\Delta S°$ = 5.1 kcal mol^{-1}; $\Delta G°$ = −12.3 kcal mol^{-1}, n = 1.00 ± 0.1). We conclude that the mutations introduced during the optimization do not affect the binding performance of the receptor.

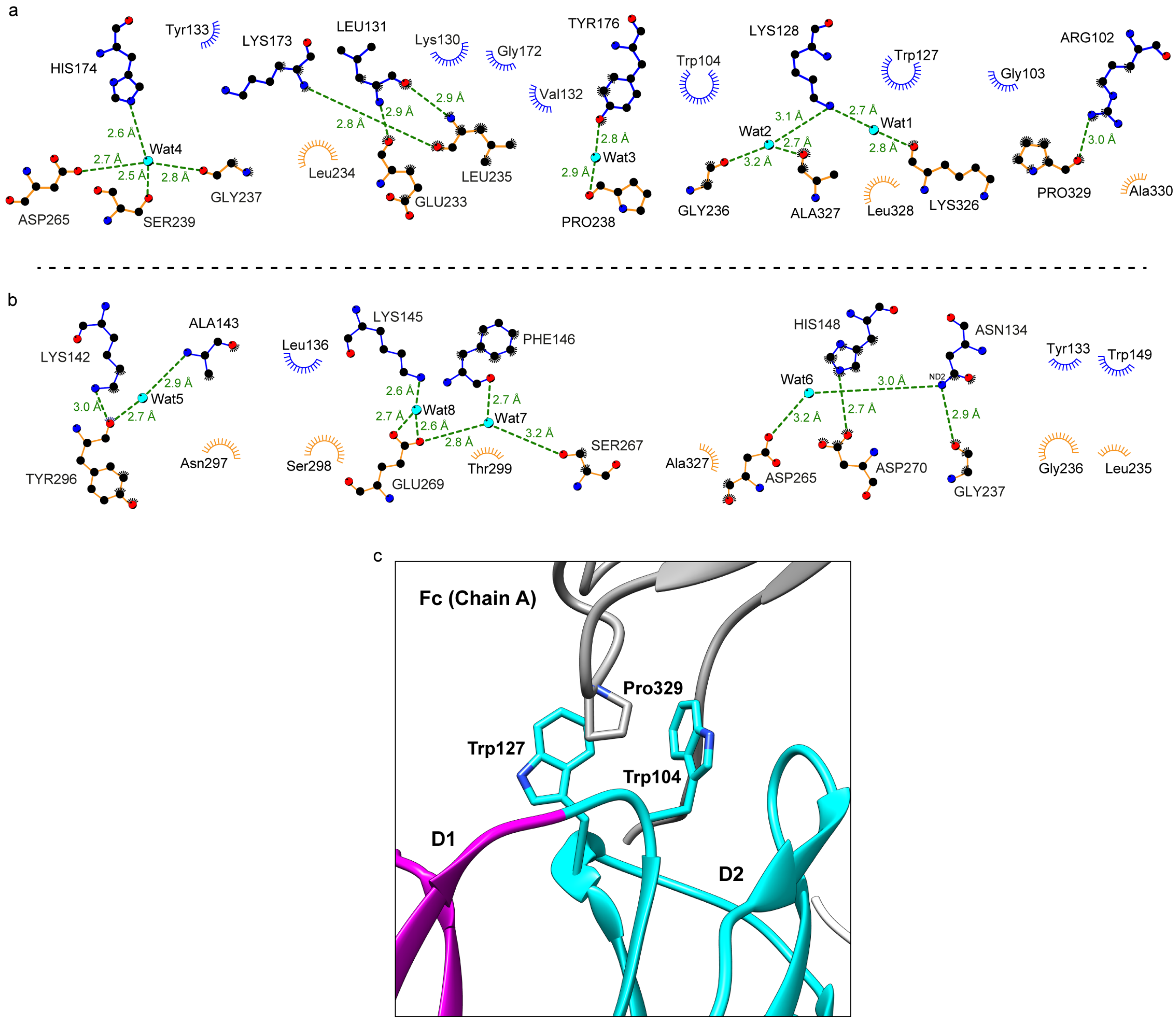

Supplementary Figure 4. Binding interface.

(a) Subsite 1. **(b)** Subsite 2. The figure was prepared with the program LigPlot[1]. In both panels hFcγRI is shown above (blue C-C bonds) and Fc below (orange C-C bonds). Bridging water molecules are also shown. The H-bonds are represented by the green dotted lines. **(c)** Close-up view of the conserved interaction between Pro329 of Fc and Trp104 and Trp127 of domain D2 of hFcγRI.

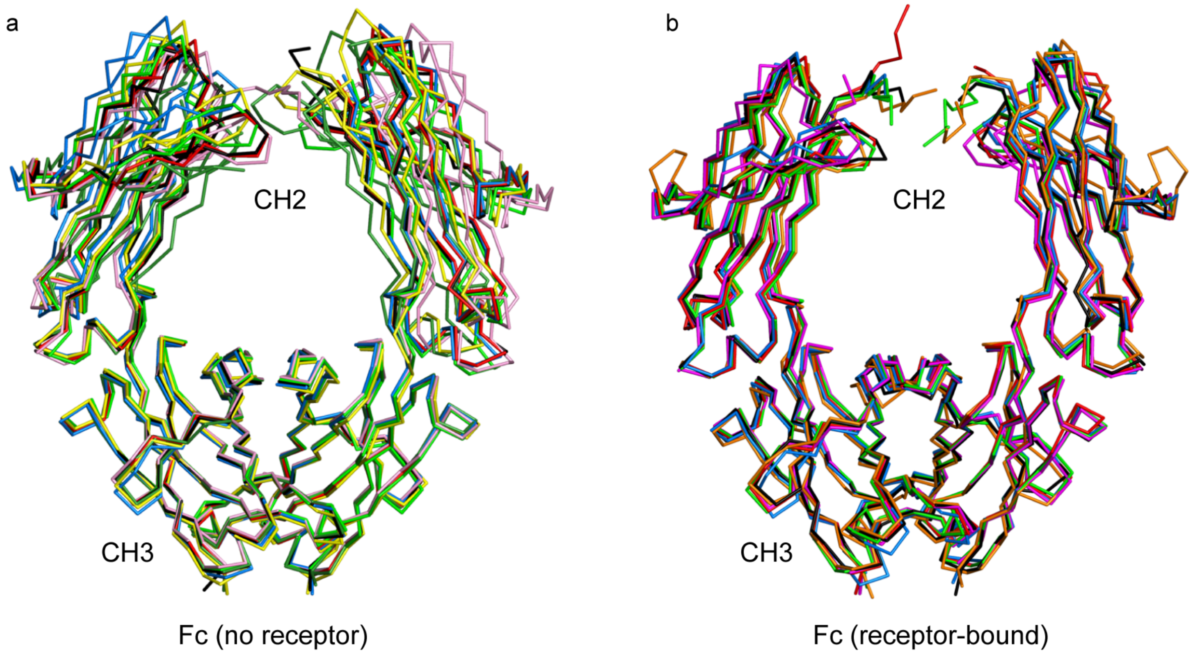

Supplementary Figure 5. Overlay of multiple crystal structures of Fc.

(a) Overlay of multiple structures of Fc in the unbound form. Only the CH3 domains are used to superimpose the structures. The most disordered chain (high B-factors) of Fc is placed on the left side. The Fc structures depicted are: this study (red), 1L6X (green), 4KU1 (marine), 4BM7 (magenta), 3AVE (black), 4Q74 (pink), 4BYH (yellow), and 4ACP (forest). The average RMSD value for CH2 and CH3 domains are 2.53 ± 1.49 Å and 0.24 ± 0.06 Å, respectively. **(b)** Overlay of Fc bound to Fcγ receptors using the CH3 domain as template. The Fcγ receptors are not shown. The Fc molecules depicted are: our study (bound to hFcγRI, red), 3AY4 (bound to hFcγRIIIa, green), 3SGK (bound to hFcγRIIIa, marine), 3WJJ (bound to hFcγRIIb, magenta), 1T83 (bound to hFcγRIIIb, black), and 1EK4 (bound to hFcγRIIIb, orange). The average RMSD value for the CH2 and the CH3 domains are 0.81 ± 0.25 Å and 0.31 ± 0.11 Å, respectively.

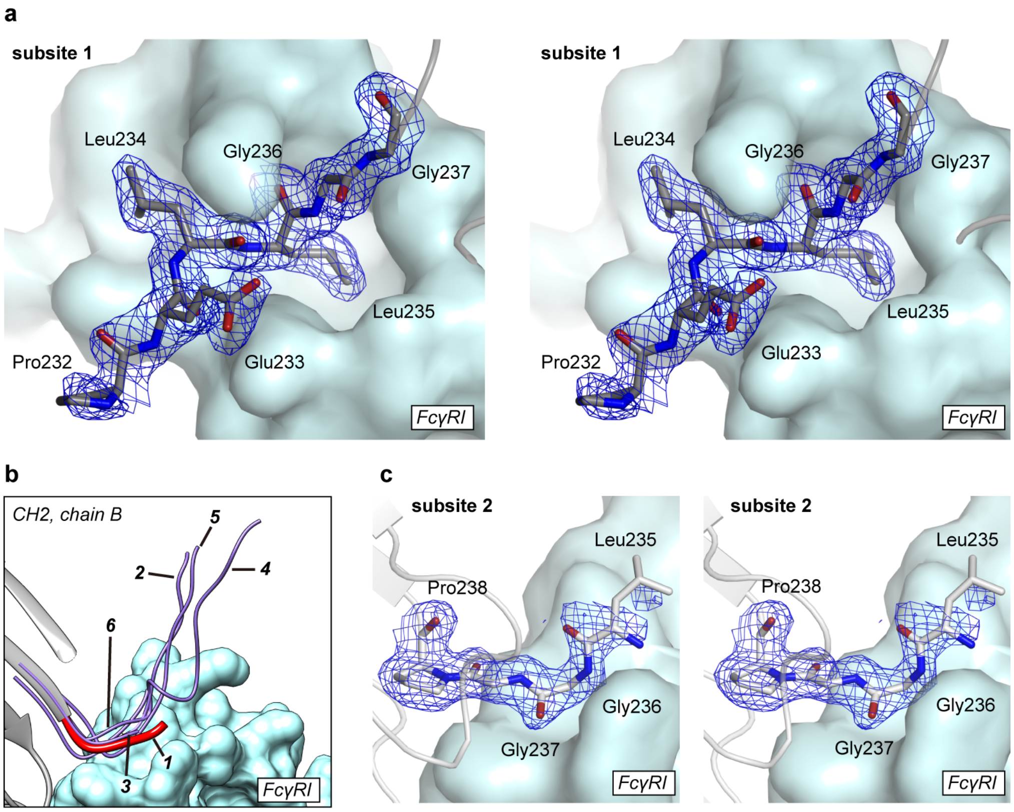

Supplementary Figure 6. Electron density of the hinge region.

(a) Stereoview of the sigmaA weighted (2Fo − Fc) electron density map of residues 232-237 of chain A of Fc in the hydrophobic pocket of subsite 1 of hFcγRI (σ = 1). The surface of hFcγRI is shown in light blue. **(b)** Comparison of the conformation of the lower hinge region of Fc in subsite 2 for various Fcγ receptors. The lower hinge region of Fc bound to hFcγRI is depicted with a red ribbon. The lower hinge region of the other structures is depicted with purple ribbons. The numbers correspond to 1, hFcγRI; 2, hFcγRIIIa (PDB code 3AY4); 3, hFcγRIIIa (PDB code 3SGK); 4, hFcγRIIIa (PDB code 3SGJ); 5, hFcγRIIIb (PDB code 1E4K); 6, hFcγRIIIb (PDB code 1T83). **(c)** Stereoview of the sigmaA weighted (2Fo − Fc) electron density map of residues 235-238 of chain B of Fc in subsite 2 of hFcγRI (σ = 1). The surface of hFcγRI is shown in light blue.

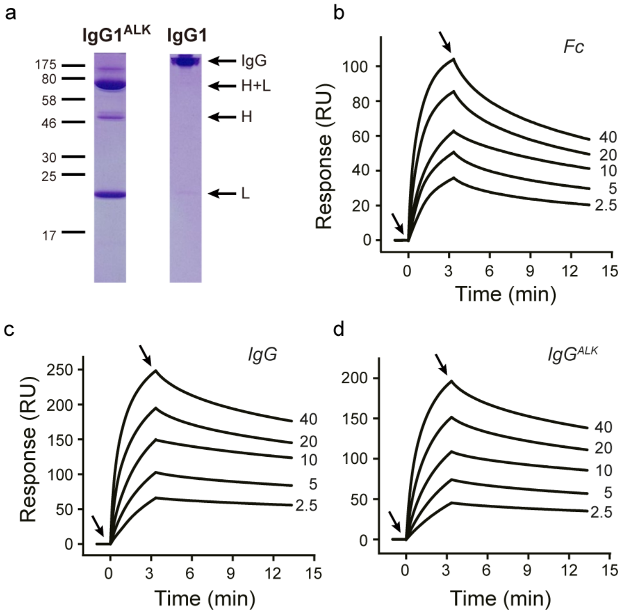

Supplementary Figure 7. Disulfide-bonds at the hinge have little influence on the binding to hFcγRI. **(a)** Non-reducing SDS-PAGE demonstrates alkylation of IgG1 using iodoacetamine. The disappearance of the band corresponding to the complete IgG1 indicates successful alkylation of the intermolecular disulphide bonds at the hinge region. A minor fraction of reduced light-chain is also observed. **(b-d)** Binding if immune molecules to hFcγRI. SPR sensorgrams correspond to **(b)** Fc, **(c)** IgG1, and **(d)** IgG1^{ALK}. The first arrow indicates injection of analyte (Fc, IgG1, or IgG1^{ALK}), and the second arrow that of buffer. Values of kinetic parameters determined for Fc were k_{on} = 7.3 × 10^{5} M^{-1}s^{-1}, k_{off} = 8.2 × 10^{-4} s^{-1}, and K_D = 1.1 nM. The values for IgG1 were k_{on} = 6.2 × 10^{5} M^{-1}s^{-1}, k_{off} = 4.1 × 10^{-4} s^{-1}, and K_D = 0.7 nM. The values for IgG1^{ALK} were k_{on} = 6.9 × 10^{5} M^{-1}s^{-1}, k_{off} = 9.6 × 10^{-4} s^{-1}, and K_D = 1.4 nM.

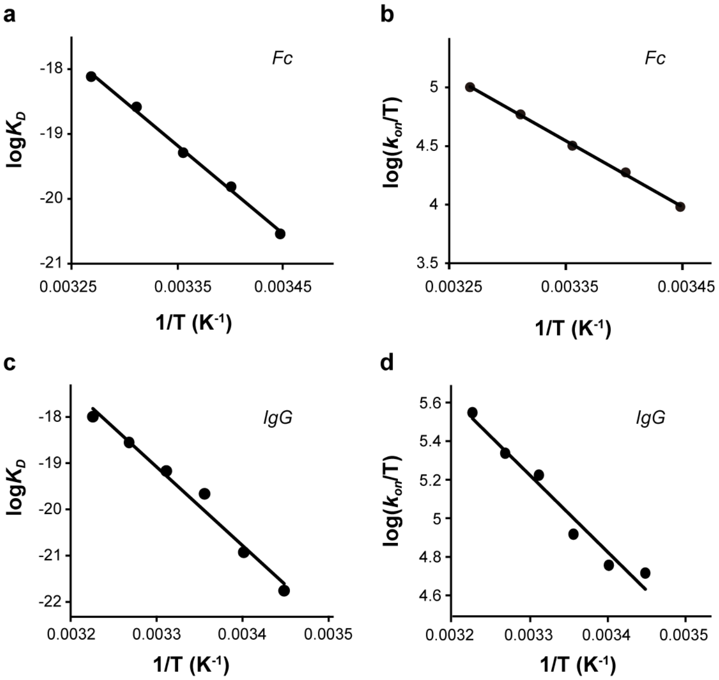

Supplementary Figure 8. Thermodynamic parameters of binding of Fc and IgG1 to hFcγRI by SPR. **(a, b)** Data corresponds to Fc. **(c, d)** Data corresponds to IgG1. The thermodynamic parameters obtained from the temperature dependence (van't Hoff approximation) were determined from panels (a) and (c). The values corresponding to Fc were K_D = 4.2 nM, $\Delta G_{vH} = -11.4$ kcal mol^{-1}, $\Delta H_{vH} = -26.8$ kcal mol^{-1}, and $-T\Delta S_{vH} = 15.4$ kcal mol^{-1}. The values determined for IgG1 were K_D = 2.9 nM, $\Delta G_{vH} = -11.9$ kcal mol^{-1}, $\Delta H_{vH} = -33.8$ kcal mol^{-1}, and $-T\Delta S_{vH} = 22.0$ kcal mol^{-1}. The thermodynamic parameters of the transition state were determined from the data represented in panels (b) and (d) using the Eyring approximation. The values corresponding to Fc were $\Delta G^{\ddagger} = 11.4$ kcal mol^{-1}, $\Delta H^{\ddagger} = 11.2$ kcal mol^{-1}, and $-T\Delta S^{\ddagger} = 0.2$ kcal mol^{-1}. The values determined for IgG1 were $\Delta G^{\ddagger} = 11.1$, $\Delta H^{\ddagger} = 7.9$ kcal mol^{-1}, and $-T\Delta S^{\ddagger} = 3.2$ kcal mol^{-1}.

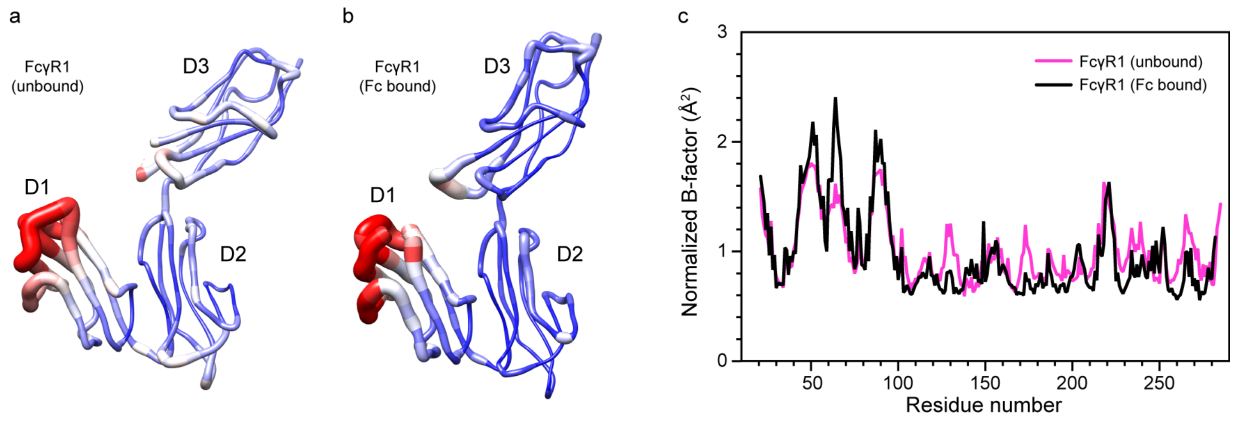

Supplementary Figure 9. B-factors of hFcγRI.

(a) Sausage representation of unbound hFcγRI colored by B-factor (red, high B-factor; blue, low B-factor). **(b)** Sausage representation of unbound hFcγRI colored by B-factor (as above). **(c)** B-factor plot of bound and unbound hFcγRI. Values are normalized with the average B-factor of each structure (81.6 Å^2 and 47.6 Å^2 for the unbound and bound receptor, respectively).

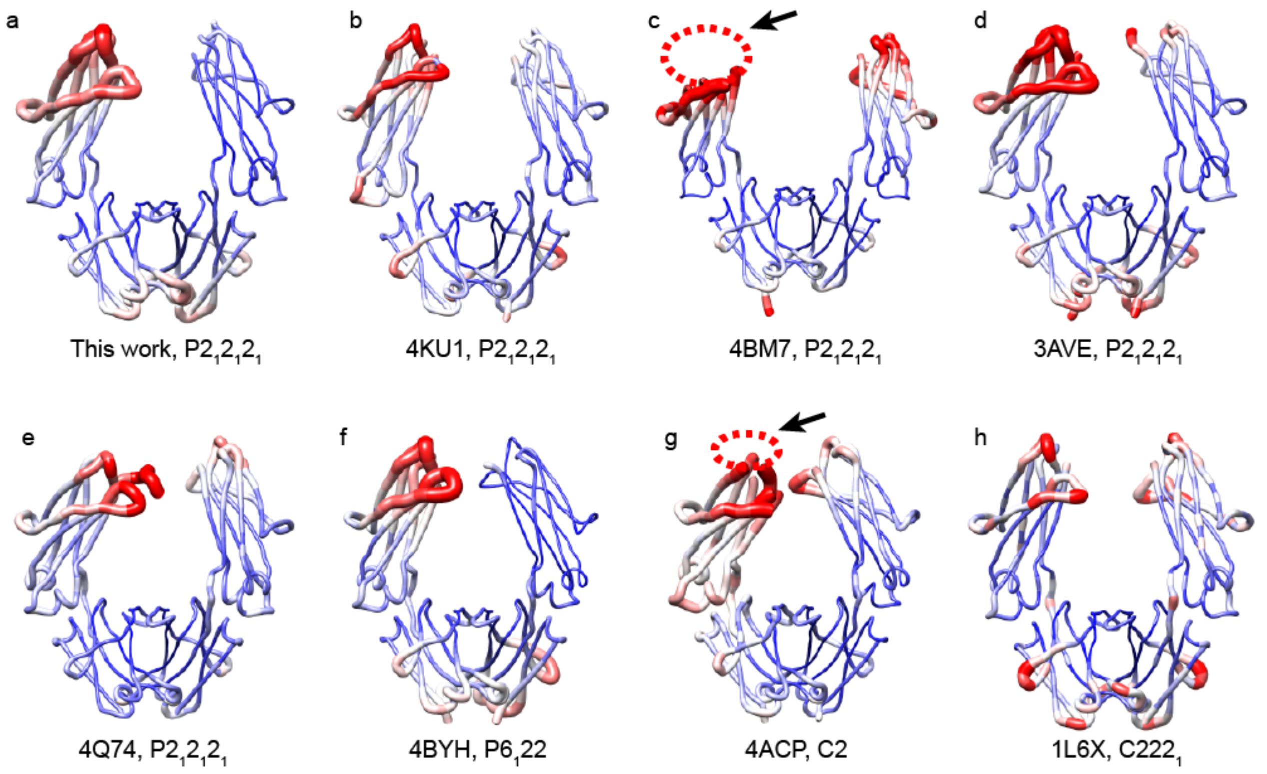

Supplementary Figure 10. Dynamic and asymmetric features of Fc.

(a-h) Sausage representation of Fc structures retrieved from the PDB (see criteria below). Red indicates high B-factor. Blue indicates low B-factor. The protein chain with higher B-factors is represented on the left side of the dimer, except that of 1L6X which is perfectly symmetric (in 1L6X Fc is bound to a fragment of protein A, not depicted). The PDB entry codes and space-groups are indicated under each structure. Only structures corresponding to unique space-groups at better than 3.0 Å resolution, or any structure at high resolution (2.2 Å resolution or better) and whose structure factors are deposited, published, and belong to Fc molecules that have not been significantly engineered are shown. We did not consider bispecific or asymmetric Fc, aptamers, monomeric Fc, etc. We note that in the crystal structures of 4BM7 and 4ACP there are several residues missing in the dynamic chain (indicated by a circle and an arrow).

Supplementary Table 1. Primary sequence of the low-hinge region of human IgG subtypes[2].

	Residue number									
**IgG subtype**	**233**	**234**	**235**	**236**	**237**	**238**	**239**	**240**	**241**	**242**
IgG1	E	L	L	G	G	P	S	V	F	L
IgG2	P	V	A	G	−	P	S	V	F	L
IgG3	E	L	L	G	G	P	S	V	F	L
IgG4	E	F	L	G	G	P	S	V	F	L

Supplementary Table 2. Interaction surface between Fc and hFcγRI.[a]

Subsite 1		Subsite 2	
**Residue**[b]	**BSA ($Å^2$)**	**Residue**[b]	**BSA ($Å^2$)**
Pro232(A)	26.0	Leu235(B)	77.9
Glu233(A)	71.0	Gly236(B)	80.9
Leu234(A)	110.7	Gly237(B)	19.2
Leu235(A)	186.0	Pro238(B)	0.3
Gly236(A)	44.8	Ser239(B)	0.5
Gly237(A)	23.5	Asp265(B)	35.7
Pro238(A)	3.2	Val266(B)	0.6
Ser239(A)	18.4	Ser267(B)	37.1
Asp265(A)	12.4	His268(B)	0.3
Asp270(A)	1.4	Glu269(B)	26.0
Lys326(A)	10.6	Asp270(B)	8.3
Ala327(A)	22.6	Glu294(B)	2.5
Leu328(A)	11.6	Gln295(B)	1.4
Pro329(A)	133.1	Tyr296(B)	60.7
Ala330(A)	23.1	Asn297(B)	44.1
Pro331(A)	14.9	Ser298(B)	61.0
Ile332(A)	4.7	Thr299(B)	27.5
GluNAc451(A)	3.2	Ala327(B)	8.2
GluNAc452(A)	4.5	GluNAc451(B)	61.3
Arg102(C)	64.3	Leu131(C)	15.1
Gly103(C)	18.6	Tyr133(C)	51.7
Trp104(C)	95.8	Asn134(C)	38.6
Leu105(C)	1.2	Leu136(C)	26.1
Ala126(C)	3.5	Tyr138(C)	3.3
Trp127(C)	62.2	Gly141(C)	7.3
Lys128(C)	13.4	Lys142(C)	66.2
Asp129(C)	2.2	Ala143(C)	27.2
Lys130(C)	76.8	Phe144(C)	8.9
Leu131(C)	61.6	Lys145(C)	27.9
Val132(C)	7.2	Phe146(C)	89.3
Tyr133(C)	15.0	Phe147(C)	0.5
Gly172(C)	19.1	His148(C)	95.6
Lys173(C)	79.0	Trp149(C)	38.1
His174(C)	43.4	Arg175(C)	3.5
Tyr176(C)	23.9		

[a]Buried surface area (BSA) was calculated in the PISA server.

[b]Chain (A) and (B) refers to Fc. Chain (C) corresponds to hFcγRI.

Supplementary Table 3. Direct and water-mediated H-bonds between Fc and hFcγRI.[a]

**Protein-Protein**			
**Residue Fc**	**Residue hFcγRI**	**Distance (Å)**	**Subsite**
Glu233 (A)	Leu131	2.9	1
Leu235 (A)	Lys173	2.8	1
Leu235 (A)	Leu131	2.9	1
Pro329 (A)	Arg102	3.0	1
Gly237 (B)	Asn134	2.9	2
Asp270 (B)	His148	2.7	2
Tyr296 (B)	Lys142	2.9	2
**Water mediated**			
**Water**	**Residue[a]**	**Distance (Å)**	**Subsite**
Water1	Lys326(A)	2.8	1
Water1	Lys128(C)	2.7	1
Water2	Gly236(A)	3.2	1
Water2	Ala327(A)	2.7	1
Water2	Lys128(C)	3.1	1
Water3	Pro238(A)	2.9	1
Water3	Tyr176(C)	2.8	1
Water4	Gly237(A)	2.8	1
Water4	Ser239(A)	2.5	1
Water4	Asp265(A)	2.7	1
Water4	His174(C)	2.6	1
Water5	Asp265(B)	3.2	2
Water5	Asn134(C)	3.0	2
Water6	Tyr296(B)	2.7	2
Water6	Ala143(C)	2.9	2
Water7	Ser267(B)	3.2	2
Water7	Glu269(B)	2.8	2
Water7	Phe146(C)	2.7	2
Water8	Glu269 OE1(B)	2.7	2
Water8	Glu269 OE2(B)	2.6	2
Water8	Lys145(C)	2.6	2

[a]Chain (A) and (B) refers to Fc. Chain (C) corresponds to hFcγRI.

Supplementary Table 4. Interaction surface between Fc and hFcγRIIa.[a,b]

Subsite 1		Subsite 2	
**Residue**[c]	**BSA (Å^2)**	**Residue**[c]	**BSA (Å^2)**
Leu234(A)	1.2	Leu234(B)	44.4
Leu235(A)	62.6	Leu235(B)	0.2
Gly236(A)	54.0	Gly236(B)	11.4
Gly237(A)	24.5	Gly237(B)	37.2
Pro238(A)	2.3	Pro238(B)	7.4
Ser239(A)	8.1	Ser239(B)	41.4
Lys326(A)	3.9	Val264(B)	13.9
Ala327(A)	20.9	Asp265(B)	51.8
Leu328(A)	21.3	Val266(B)	33.8
Pro329(A)	102.9	Ser267(B)	23.2
Ala330(A)	7.0	His268(B)	1.7
Ile332(A)	1.7	Asn297(B)	57.4
Gln21(C)	13.7	Ser298(B)	30.8
Gln89(C)	16.0	Thr299(B)	6.8
Trp90(C)	82.7	Val119(C)	39.5
Trp113(C)	65.2	Lys120(C)	64.7
Lys116(C)	14.4	Thr122(C)	4.7
Pro117(C)	36.2	Phe124(C)	2.8
Ile158(C)	25.1	Ser129(C)	9.6
Gly159(C)	19.7	Lys131(C)	0.3
Thr161(C)	8.5	Phe132(C)	63.6
Phe163(C)	2.7	Arg134(C)	102.7
		Leu135(C)	21.1.
		Asn157(C)	5.2
		Gly159(C)	10.8
		Tyr160(C)	100.7

[a]BSA was calculated in the PISA server.

[b]PDB entry code is 3RY6. These data should be taken with care because the resolution is very low (3.8 Å).

[c]Chain (A) and (B) refers to Fc. Chain (C) corresponds to hFcγRIIa (PDB entry code is 3RY6).

Supplementary Table 5. H-bonds between Fc and hFcγRIIa.[a]

Residue Fc	Residue hFcγRI	Distance (Å)	Subsite
Val266(B)	Arg134	2.9	2

[a]PDB entry code is 3RY6. The data should be taken with care because the resolution is very low (3.8 Å).

Supplementary Table 6. Interaction surface between Fc and hFcγRIIb.[a,b]

Subsite 1		Subsite 2	
**Residue[b]**	**BSA (Å^2)**	**Residue[b]**	**BSA (Å^2)**
Glu233(B)	17.7	Asp265(A)	29.8
Leu234(B)	0.1	Val266(A)	3.9
Leu235(B)	8.5	Ser267(A)	50.8
Gly236(B)	20.7	His268(A)	4.5
Gly237(B)	29.5	Glu269(A)	22.3
Pro238(B)	41.9	Asp270(A)	13.9
Lys326(B)	5.2	Tyr296(A)	11.8
Ala327(B)	20.7	Asn297(A)	29.2
Leu328(B)	11.0	Ser298(A)	72.1
Pro329(B)	129.7	Thr299(A)	11.8
Ala330(B)	34.2	Asn325(A)	4.1
Ile332(B)	9.7	Ala327(A)	29.9
Gln18(C)	14.0	Pro329(A)	0.2
Ser85(C)	37.0	GluNAc1001(A)	47.6
Glu86(C)	10.2	Lys117(C)	8.3
Trp87(C)	79.7	Thr119(C)	11.1
Trp110(C)	55.3	Phe121(C)	3.1
Lys111(C)	5.5	Ser126(C)	45.6
Lys113(C)	20.3	Lys127(C)	6.6
Gly156(C)	0.2	Lys128(C)	18.5
Tyr157(C)	5.7	Phe129(C)	112.9
Thr158(C)	61.9	Ser130(C)	6.7
Leu159(C)	1.7	Arg131(C)	105.3
Tyr160(C)	19.5		

[a]BSA was calculated with the PISA server.

[b]Chain (A) and (B) refers to a modified version of Fc containing mutations in the critical hinge region. Chain (C) corresponds to FcγRIIb (PDB entry code is 3WJJ).

Supplementary Table 7. H-bonds between Fc and hFcγRIIb.[a]

Residue Fc	Residue hFcγRIIb	Distance (Å)	Subsite
Gly237(B)	Tyr160	3.1	1
Ser298(A)	Ser126	3.1	2
Asp270(A)	Arg131	3.1	2
Asp270(A)	Arg131	2.8	2
Asp270(A)	Arg131	2.9	2
Asp270(A)	Arg131	2.8	2

[a]PDB entry code is 3WJJ. We note the critical hinge region of Fc has been mutated in this structure.

Supplementary Table 8. Interaction surface between Fc and hFcγRIIIa.[a]

Subsite 1		Subsite 2	
**Residue**[b]	**BSA (Å^2)**	**Residue**[b]	**BSA (Å^2)**
Glu233(B)[c]	0.2	Leu234(A)[c]	0.4
Leu235(B)	103.0	Leu235(A)[c]	46.1
Gly236(B)	60.9	Gly236(A)	75.4
Gly237(B)	1.9	Gly237(A)	22.0
Pro238(B)	4.2	Pro238(A)	2.1
Ser239(B)	12.6	Ser239(A)	11.7
Asp265(B)[c]	2.4	Asp265(A)	39.5
Asp270(B)[c]	1.3	Ser267(A)	38.8
Lys326(B)	15.9	His268(A)	6.3
Ala327(B)	21.2	His269(A)	31.9
Leu328(B)	12.1	Asp270(A)	1.1
Pro329(B)	122.0	Glu294(A)[c]	2.2
Ala330(B)	21.2	Gln295(A)[c]	0.7
Ile332(B)	7.9	Tyr296(A)	78.6
Glu21(C)	6.0	Asn297(A)	44.7
Lys22(C)[c]	4.9	Ser298(A)	52.1
Ile88(C)	35.8	Thr299(A)	23.6
Gly89(C)	16.4	Asn325(A)	0.5
Trp90(C)	49.9	Ala327(A)	18.6
Trp113(C)	63.3	GluNAc1001(A)	54.4
Lys114(C)	8.7	His119(C)	30.8
Thr116(C)	21.7	Lys120(C)	77.4
Ala117(C)	22.1	Thr122(C)	9.8
His119(C)	3.2	LEU124(C)[c]	0.3
Val158(C)	18.9	Asn126(C)[c]	1.7
Gly159(C)	25.9	Gly127(C)	13.7
Ser160(C)[c]	2.5	Lys128(C)	58.3
Lys161(C)	76.9	Gly129(C)	34.8
		Arg130(C)	8.1
		Lys131(C)	36.6
		Tyr132(C)	83.1
		Phe133(C)	3.6
		His134(C)	108.5
		His135(C)	21.2
		Arg155(C)	13.5
		Leu157(C)[c]	3.1
		Gly159(C)[c]	14.0

[a]BSA was calculated in the PISA server.

[b]Chain (A) and (B) refers to Fc. Chain (C) corresponds to hFcγRIIIa (PDB entry codes are 3AY4 and 3SGK).

[c]Interaction observed only in one crystal structure.

Supplementary Table 9. H-bonds between Fc and hFcγRIIIa.[a]

Residue Fc	Residue hFcγRIIIa	Distance (Å)	Subsite
Gly236(B)[b]	Lys161	2.7	1
Pro238(B)	Lys161	2.7	1
Asp265(A)	Lys120	2.6	2
Ser239(A)	Lys120	2.7	2
Gly237(A)[b]	Lys120	2.7	2
Tyr296(A)	Lys128	2.8	2
Tyr296(A)[b]	Gly129	3.1	2
Glu269(A)[b]	Lys131	2.6	2
Asp265(A)	His134	3.0	2

[a]PDB entry codes are 3AY4 and 3SGK.

[b]Interaction observed in only one crystal structure.

Supplementary Table 10. Interaction surface between Fc and hFcγRIIIb.[a]

Subsite 1		Subsite 2	
**Residue**[b]	**BSA (Å^2)**	**Residue**[b]	**BSA (Å^2)**
Glu233(B)[c]	5.1	Leu234(A)[c]	28.3
Leu234(B)	3.8	Leu235(A)	47.9
Leu235(B)	95.7	Gly236(A)	46.6
Gly236(B)	45.1	Gly237(A)	33.3
Gly237(B)	11.9	Pro238(A)	2.3
Pro238(B)	2.8	Ser239(A)	10.6
Ser239(B)	15.0	Asp265(A)	36.0
Asp265(B)[c]	3.7	Val266(A)[c]	0.5
Lys326(B)	10.4	Ser267(A)	29.8
Ala327(B)	15.1	His268(A)[c]	4.1
Leu328(B)	13.2	His269(A)	17.1
Pro329(B)	124.7	Asp270(A)[c]	0.2
Ala330(B)	17.1	Tyr296(A)[c]	44.7
Ile332(B)	9.5	Asn297(A)	29.7
Glu21(C)	3.7	Ser298(A)	62.1
Lys22(C)[c]	0.8	Thr299(A)	21.1
Ile88(C)	34.1	Asn325(A)[c]	0.2
Gly89(C)	13.8	Ala327(A)	15.8
Trp90(C)	50.4	Pro329(A)[c]	3.9
Trp113(C)	57.7	GluNAc448(A)	76.9
Thr116(C)	19.6	GluNAc448(A)[c]	5.8
Ala117(C)	20.3	His119(C)	40.9
His119(C)	2.1	Lys120(C)	81.2
Val158(C)	15.9	Thr122(C)	3.6
Gly159(C)	32.8	Gly127(C)[c]	6.9
Ser160(C)	13.5	Lys128(C)[c]	19.8
Lys161(C)	73.8	Asp129(C)	52.5
		Arg130(C)	5.5
		Lys131(C)	23.6
		Tyr132(C)	78.6
		Phe133(C)[c]	0.3
		His134(C)	105.6
		His135(C)	40.2
		Arg155(C)[c]	4.9
		Leu157(C)[c]	9.1
		Gly159(C)	6.7
		Ser160(C)	11.3

[a]BSA was calculated in the PISA server.

[b]Chain (A) and (B) refers to Fc. Chain (C) corresponds to hFcγRIIIb (PDB entry codes are 1T83 and 1E4K).

[c]Interaction observed only in one crystal structure.

Supplementary Table 11. H-bonds between Fc and hFcγRIIIb.[a]

Residue Fc	Residue hFcγRIIIb	Distance (Å)	Subsite
Gly236(B)[b]	Lys161	2.9	1
Pro238(B)[b]	Lys161	2.7	1
Asp265(A)[b]	Lys120	3.1	2
Ser239(A)	Lys120	3.0	2
Leu235(A)[b]	His135	3.0	2
Asp265(A)[b]	His134	3.1	2

[a]PDB entry codes are 1T83 and 1E4K.

[b]Interaction observed in only one crystal structure.

Supplementary Note 1.

Primary sequence of human FcγRI

^{16}QVDTTKAVITLQPPWVSVFQEETVTLHCEVLHLPGSSSTQWFLNGTATQTSTPSYRITSASVNDS
GEYRCQRGLSGRSDPIQLEIHRGWLLLQVSSRVFTEGEPLALRCHAWKDKLVYNVLYYRNGKAF
KFFHWNSNLTILKTNISHNGTYHCSGMGKHRYTSAGISVTVKELFPAPVLNASVTSPLLEGNLVTL
SCETKLLLQRPGLQLYFSFYMGSKTLRGRNTSSEYQILTARREDSGLYWCEAATEDGNVLKRSPE
LELQVLGLQLPTPV^{289}HHHHHH

Primary sequence of optimized human FcγRI

^{16}QVDTPKAVIKLQPPWVSVFQEESVTLHCEVPHLPGSSSTQWFLNGTAIQTSTPTYHITSASEDDS
GEYRCQRGLSGRSDPIQLEVHRGWLLLQVSSRVLTEGEPLALRCHAWKDKLVYNVLYYRNGKA
FKFFHWNSNLTILKTNMSHSGTYHCSGMGKHRYTSAGISVTVKELFPAPVLTASVTSPLLEGTPV
TLSCETKLLLQRPGLQLYFSFYMGSKTLRGRDTSSEYQILTARREDSGLYWCEAATEDGNVLKRS
PELELQVLGHQQPTPV^{289}HHHHHH

Supplementary References

1. Laskowski, R.A. & Swindells, M.B. LigPlot+: Multiple Ligand-Protein Interaction Diagrams for Drug Discovery. *J. Chem. Inf. Model.* **51**, 2778-2786 (2011).
2. Radaev, S. & Sun, P. Recognition of immunoglobulins by Fc gamma receptors. *Mol. Immunol.* **38**, 1073-1083 (2002).
